# Uncoupling of Molecular Maturation from Peripheral Target Innervation in Nociceptors Expressing a Chimeric TrkA/TrkC Receptor

**DOI:** 10.1371/journal.pgen.1004081

**Published:** 2014-02-06

**Authors:** Svetlana Gorokhova, Stéphane Gaillard, Louise Urien, Pascale Malapert, Wassim Legha, Grégory Baronian, Jean-Pierre Desvignes, Serge Alonso, Aziz Moqrich

**Affiliations:** 1 Aix-Marseille-Université, CNRS, Institut de Biologie du Développement de Marseille, UMR 7288, Marseille, France; 2 MGX-Montpellier GenomiX, c/o Institut de Génomique Fonctionnelle, Montpellier, France; University of California San Diego, United States of America

## Abstract

Neurotrophins and their receptors control a number of cellular processes, such as survival, gene expression and axonal growth, by activating multiple signalling pathways in peripheral neurons. Whether each of these pathways controls a distinct developmental process remains unknown. Here we describe a novel knock-in mouse model expressing a chimeric TrkA/TrkC (TrkAC) receptor from *TrkA* locus. In these mice, prospective nociceptors survived, segregated into appropriate peptidergic and nonpeptidergic subsets, projected normally to distinct laminae of the dorsal spinal cord, but displayed aberrant peripheral target innervation. This study provides the first *in vivo* evidence that intracellular parts of different Trk receptors are interchangeable to promote survival and maturation of nociceptors and shows that these developmental processes can be uncoupled from peripheral target innervation. Moreover, adult homozygous TrkAC knock-in mice displayed severe deficits in acute and tissue injury-induced pain, representing the first viable adult Trk mouse mutant with a pain phenotype.

## Author Summary

Sensory neurons located in dorsal root ganglia are critical for perception of various stimuli by transmitting information from their peripheral targets to the spinal cord. During embryonic development, distinct populations of sensory neurons are defined based on expression of neurotrophin receptors Trks. Pain and temperature sensing neurons, or nociceptors, express NGF receptor TrkA, which control a number of diverse developmental processes, such as survival, gene expression and skin innervation. How these distinct processes are regulated by activation of same Trk receptor is currently unknown. Using a knock in approach, we generated a mouse with nociceptive neurons expressing a modified TrkA/TrkC receptor, which responds to NGF but signals through the intracellular part of another neurotrophin receptor, TrkC. Contrary to all previously reported NGF and TrkA mutants, these mice were viable and exhibited no obvious defects. Surprisingly, nociceptive neurons from these mice survived and matured normally, but failed to correctly innervate their peripheral target, skin. Thus, the intracellular parts of highly related receptors TrkA and TrkC are interchangeable for support of certain developmental processes but not others. Moreover, adult TrkA/TrkC mice exhibited drastic defects in pain sensation, making it an excellent model to study the role of NGF in nociception.

## Introduction

Sensory neurons of the dorsal root ganglia (DRG) represent a powerful model for studying neuronal survival, fate determination and neuronal circuit assembly during development. Indeed, after initial fate specification, these neurons establish connections with neurons in the spinal cord and with the appropriate targets in the periphery, such as various structures in the skin or muscle. Extracellular cues that are encountered by a developing sensory neuron, both en route and in the final destination tissue, then in turn control its survival and maturation. Neurotrophins (NTs) and their receptors (Trk) play key roles in this intricately balanced dialog between growing axons and their surroundings by controlling multiple aspects of development.

Neurotrophin NGF controls survival of nociceptors, which are pain and temperature sensing neurons expressing NGF receptor TrkA during development and projecting to dorsal spinal cord centrally and to skin in the periphery [1], [2]. Indeed, mice lacking TrkA or NGF exhibit massive apoptosis of these neurons as early as embryonic day 13.5 [3]. However, when survival of nociceptors from mice lacking NGF or TrkA was rescued by concomitant deletion of a pro-apoptotic protein Bax, these neurons failed to express such nociceptor-specific protein markers as CGRP and substance P, suggesting that NGF/TrkA signaling also controls gene expression in nociceptors [4]. Moreover, the postnatal maturation of nociceptive neurons also requires NGF signaling cascade [5].

In addition to controlling survival and maturation of cutaneous nociceptors, NGF is critical for axonal extension and peripheral target innervation by these neurons. Indeed, neurites from DRG explants grew towards NGF source *in vitro* [6] and beads containing NGF or other neurotrophins directed growth of sensory nerves in slice cultures of mouse embryos [7]. Moreover, after initially normal axonal extension, nociceptive neurons from NGF/Bax double mutants failed to branch and innervate the epidermis normally [4], [8].

Finally, it has been shown that expression of NT3 receptor TrkC from *TrkA* locus engaged a subset of former TrkA nociceptors to become TrkC-expressing proprioceptors [9]. Did these neurons switch fate because they responded to NT3 instead of NGF or because they were lacking intracellular TrkA signaling to confer the nociceptive fate? How can downstream signaling pathways activated by the same ligand/receptor complex, NGF/TrkA, control such distinct outcomes as survival, cell fate acquisition, maturation and target innervation? In order to answer these questions, we generated knock-in mice expressing a chimeric receptor TrkAC, composed of the extracellular part of TrkA and the intracellular part of TrkC, from *TrkA* locus. As result, we show for the first time that intracellular parts of Trk receptors are interchangeable *in vivo* to control a number of NGF/TrkA-dependent processes, such as survival, fate acquisition and postnatal maturation of nociceptive neurons. Moreover, we find that NGF-dependent survival and fate determination can be completely uncoupled from target innervation. Finally, our study describes the first viable mouse model with perturbed NGF/TrkA signalling that leads to severe deficits in pain sensation.

## Results

### Replacing TrkA with TrkAC supports survival of nociceptive neurons

To distinguish between the influence of extracellular cues and intracellular developmental programs in neurotrophin-dependent survival, cell fate specification and neuronal circuit establishment of nociceptors, we generated a mouse line expressing a chimeric TrkA/TrkC receptor (TrkAC) from *TrkA* (*Ntrk1*) locus. In these mice, the extracellular part of the chimeric Trk receptor is encoded by endogenous *TrkA* (*Ntrk1*) (exons 1–10), while the transmembrane and intracellular parts are encoded by *TrkC* (*Ntrk3*) cDNA (Figure S1A–D), thus leaving a large portion of the *Ntrk1* locus intact in order to maximize the expression of the transgene. Unlike previously generated NGF and TrkA mutant mice [1], [2], [4], [9], homozygous *TrkA^TrkAC/TrkAC^* (*TrkAC-KI*) mice survived till adulthood, were fertile and exhibited no obvious deficits, indicating that a functional Trk receptor is expressed from the *TrkA* locus.

**Figure 1:**
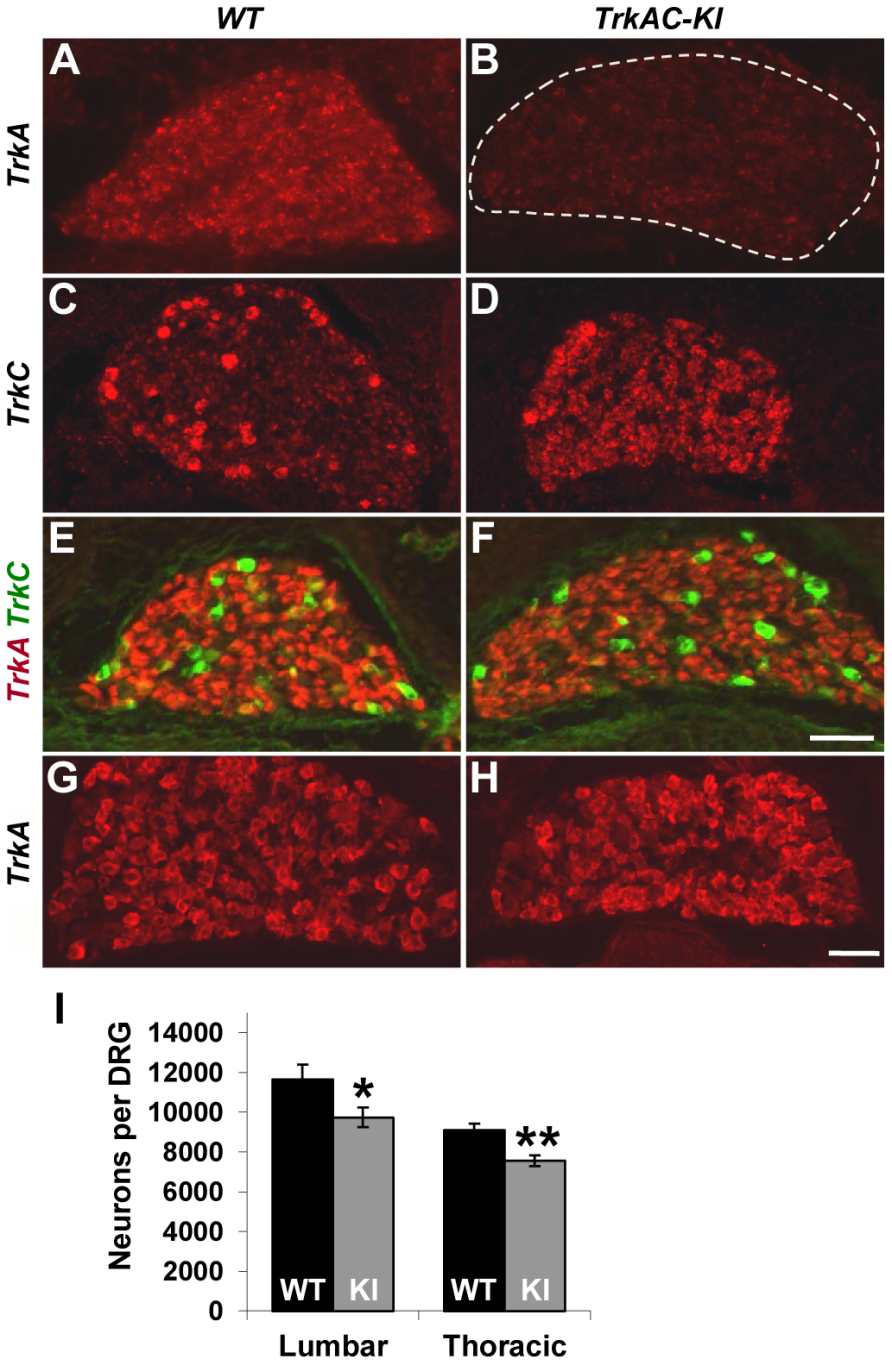
Replacing *TrkA* with *TrkAC* is compatible with grossly normal survival of sensory neurons in *TrkAC-KI* mice. (A,B) An *in situ* probe recognizing endogenous *TrkA*, but not chimeric *TrkAC* is labeling the majority of wild type E15.5 DRG neurons, while mutant DRGs lack any staining. (C,D) An *in situ* probe specific to both *TrkC* and chimeric *TrkAC* is labeling few TrkC-positive neurons in wild type embryos and the majority of neurons in the E15.5 mutant DRGs. (E,F) Immunostaining with a TrkA antibody recognizing both TrkA and TrkAC (red) and a TrkC-specific antibody (green) showing normal pattern of TrkC expression and comparable TrkA antibody immunoreactivity in E15.5 mutant DRG. (G,H) There is normal TrkA antibody immunoreactivity in DRGs from P0 *TrkAC-KI* mutant mice. (I) Total number of lumbar (L3–4) and thoracic (T11–12) DRG neurons is reduced by 16% and 18% respectively in *TrkAC-KI* mice. Data represent mean ± s.e.m. Lumbar counts: 9 mutant and 8 wild type DRGs from 4 animals for each genotype, p = 0.041; thoracic counts: 8 mutant and 6 wild type DRGs from 3 and 2 animals respectively, p = 0.0019. * p<0.05, ** p<0.01. Scale bar is 50 µm.

We first verified that endogenous TrkA is completely replaced with TrkAC chimeric receptor in *TrkAC-KI* mice. In embryonic DRGs, TrkA-positive neurons are of small diameter and represent the vast majority of DRG neurons, while TrkC neurons are of large diameter and are small-numbered. *In situ* hybridization labeling with a probe specific to endogenous *TrkA*, but not to *TrkAC*, demonstrated lack of staining in E15.5 DRGs from *TrkAC-KI* animals, while staining the majority of DRG neurons from wild type littermates (control) (Figures 1A and B). A probe specific to the *mRNA* region corresponding to the intracellular part of TrkC labelled only *TrkC* expressing neurons in control DRGs, while showing a *TrkA*-like staining pattern in addition to that of *TrkC* in *TrkAC-KI* DRGs (Figures 1C and 1D). *TrkA*-expressing neurons are completely absent by this stage in mice lacking *TrkA*, causing drastic decrease of DRG size due to 75% loss of all DRG neurons by E15.5 [3]. Remarkably, DRGs from *TrkAC-KI* mice appeared normal, suggesting that survival of neurons expressing *TrkAC* instead of *TrkA* was not affected.

We then tested TrkAC expression during several developmental time points. Immunofluorescent labeling with an antibody recognizing the extracellular part of TrkA, the common part of both TrkA and TrkAC receptors, showed a similar level of immunoreactivity of extracellular part of TrkA in control and TrkAC in *TrkAC-KI* DRGs at embryonic stages (Figures 1E, 1F) and at birth (Figures 1G and 1H). Consistent with the normal appearance of DRGs during embryonic development, the total number of sensory neurons in adult DRGs was only modestly reduced (Figures 1I).

TrkA receptor is known to be present in different glycosylation forms in a cell, but only the mature (140 kD) form is targeted to the plasma membrane [10], [11]. We found that this mature form of TrkAC chimeric protein was present in DRGs from *TrkAC-KI* embryos (Figure S1E) Further biochemical characterization of the chimeric TrkAC receptor is depicted in figures S1F–H.To evaluate the ability of TrkAC receptor to transduce NGF-induced signals, lysates from three independent NGF-stimulated E14.5 DRG cultures were electrophoresed and probed with antibodies against TrkA, phospho-Akt, phospho-ERK (MAPK) and ERK (Figure S2A). Despite lower amounts of TrkAC protein in the cultured neurons, levels of phospho-ERK reached control levels after 5 min stimulation with 100 ng/ml NGF (Figures S2B and C), suggesting that the intracellular part of TrkAC chimeric protein is more efficient at activating this effectors than the intracellular part of TrkA. Another intriguing difference between these two receptors was the higher level of baseline Akt (but not ERK) activation in DRG neurons from *TrkAC-KI* embryos (Figure S2D). These results are consistent with the previously reported finding that TrkC signaling activates Akt more strongly than TrkA signalling [12]. These data show that TrkAC receptor is able to activate downstream signaling and suggest that this chimeric receptor could promote distinct outcomes in response to NGF stimulation in comparison to TrkA. Taken together, these data showed that despite certain differences in response to NGF stimulation, TrkAC receptor is able to activate downstream effectors that are sufficient to support normal survival of nociceptive neurons during development and through adulthood.

### TrkAC induces normal segregation of nociceptors into peptidergic and nonpeptidergic neurons

**Figure 2:**
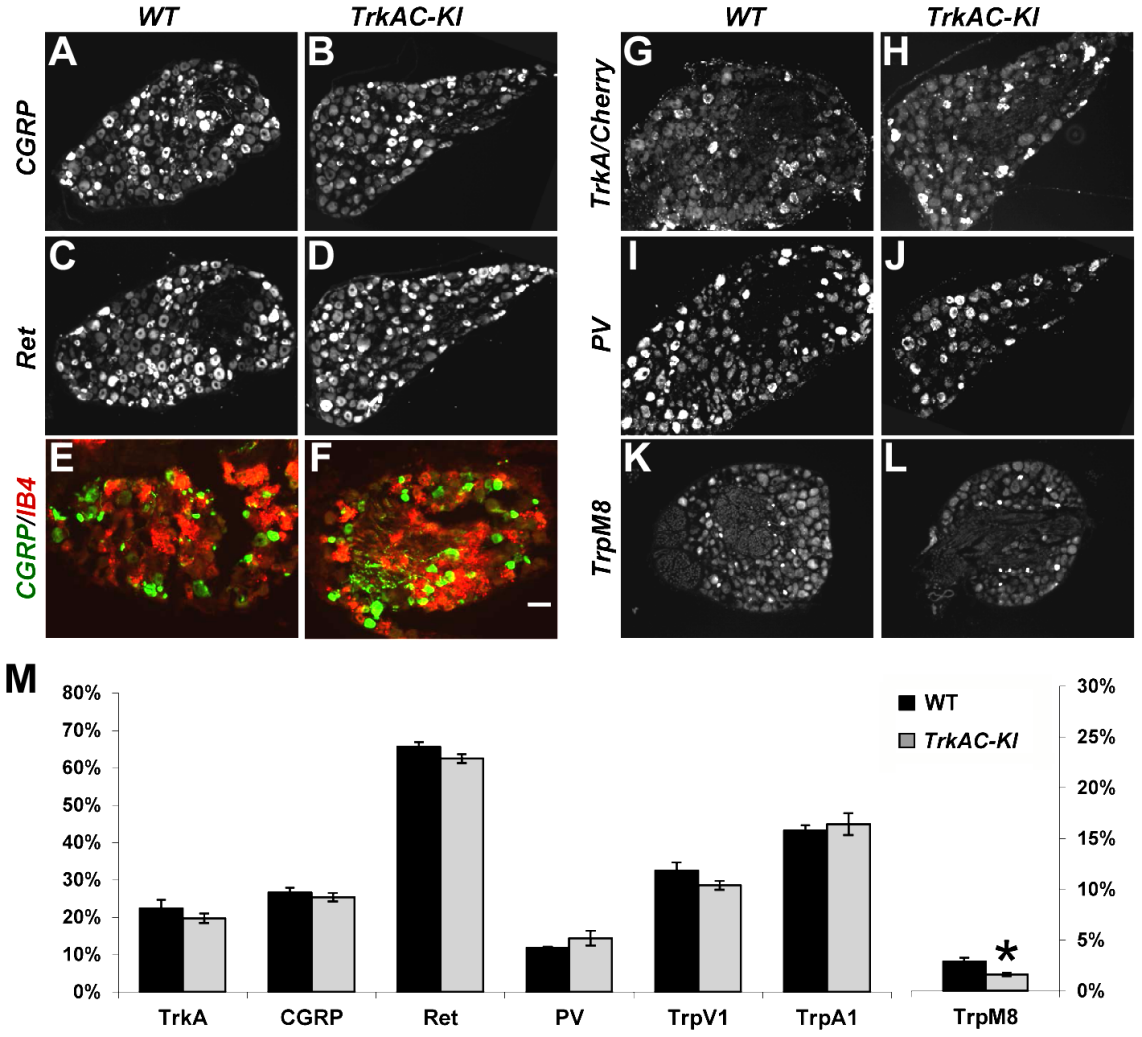
Postnatal maturation of nociceptive neurons is grossly normal in DRGs from adult *TrkAC-KI* mice. (A–D) CGRP and Ret immunostaining in lumbar DRG from adult *TrkAC-KI* and control mice show normal expression of peptidergic (A,B) and nonpeptidergic(C,D) markers in nociceptive neurons. n = 8–9 DRGs from 4 animals from each genotype. (E,F) Double fluorescent labeling with CGRP antibody and IB4 binding on adult thoracic DRGs from *TrkAC-KI* and wild type mice shows that segregation of peptidergic (CGRP) and nonpeptidergic(IB4) marker expression occurs normally in mutant mice. (G–J) *In situ* hybridization with *TrkA* (for wild type 5 DRGs from 4 animals) and *Cherry* (for *TrkAC-KI* 7 DRGs from 4 animals), as well as *PV* probes (8 mutant and 7 wild type DRGs from 4 and 3 animals respectively) on adult lumbar DRGs from *TrkAC-KI* and control animals show that fate specification of nociceptive (G,H) and proprioceptive (I,J) neurons is unaffected. (K,L) Expression of *TrpM8* mRNA in adult thoracic DRGs from *TrkAC-KI* and control mice (7 DRGs from 3 mutant and 2 wild type animals). (M) Quantification of the percentage of neurons expressing indicated markers in (A–L and Figure S3, TrpA1 and TrpV1, 4–6 DRGs from 3 mutant and 2 wild type mice). Data represent mean ± s.e.m, * p<0.05. Scale bar is 50 µm.

Several recent studies have given new insights into the molecular control of nociceptive neuron maturation [5], [13]–[16]. These neurons, specialized in sensing a myriad of noxious thermal, mechanical and chemical stimuli, are classically divided into two major subtypes: peptidergic and nonpeptidergic neurons. Each of these two categories of neurons can be further subdivided into multiple subsets according to the sets of genes they specifically express. While all prospective nociceptors express TrkA during development, only peptidergic neurons continue expressing TrkA in adulthood, while nonpeptidergic neurons downregulate TrkA in early postnatal period and express GDNF receptor Ret [5], [17]. NGF is critical for both initial expression of multiple nociceptive markers and for postnatal maturation of nociceptors [4], [5]. To determine whether replacing the intracellular part of TrkA with that of TrkC would interfere with the maturation of prospective nociceptors, we analysed the expression patterns of a battery of nociceptive and proprioceptive markers in DRGs from adult mice. Quantification of CGRP and Ret positive neurons showed no difference between *TrkAC-KI* and control DRGs, suggesting that these neurons segregated normally into peptidergic and nonpeptidergic subsets (Figures 2A–D and M). Consistent with these results, nonpeptidergic marker IB4 was appropriately excluded from the peptidergic (CGRP-positive) subpopulation of nociceptors in mutant DRGs (Figures 2E and 2F). Expression of multiple other markers, with exception of *TrpM8*, was also normal in adult *TrkAC-KI* mice (Figures 2K–M and S3). Interestingly, while expression of many NGF-dependent genes, including *GFRalpha1,2 and 3*, RUNX1, *TrpC3*, *MrgA1* and *MrgA3* is completely abolished in developing and P0 DRGs from NGF/Bax double knockout mice [5], expression of these markers was not affected in T*rkAC-KI* DRGs (Figures S4A–P and data not shown).

It has been previously shown that replacing endogenous TrkA with TrkC makes a subpopulation of former nociceptors to switch fate and become proprioceptors, leading to presence of increased numbers of PV positive neurons in DRGs [9]. We therefore quantified the number of neurons expressing parvalbumin (PV), a proprioceptive marker, as well as TrkA (or TrkAC) neurons in DRGs from control and *TrkAC-KI* mice. There was no difference, suggesting that TrkAC-expressing neurons retained their nociceptive fate (Figures 2I, 2J and 2M). Thus, replacing the intracellular part of TrkA with that of TrkC, not only promoted survival of most presumptive nociceptors, but also activated genetic programs that engaged these neurons towards normal nociceptive molecular fates, followed by normal postnatal maturation of these neurons.

### Peripheral, but not central, projections are drastically reduced in *TrkAC-KI* mice

**Figure 3:**
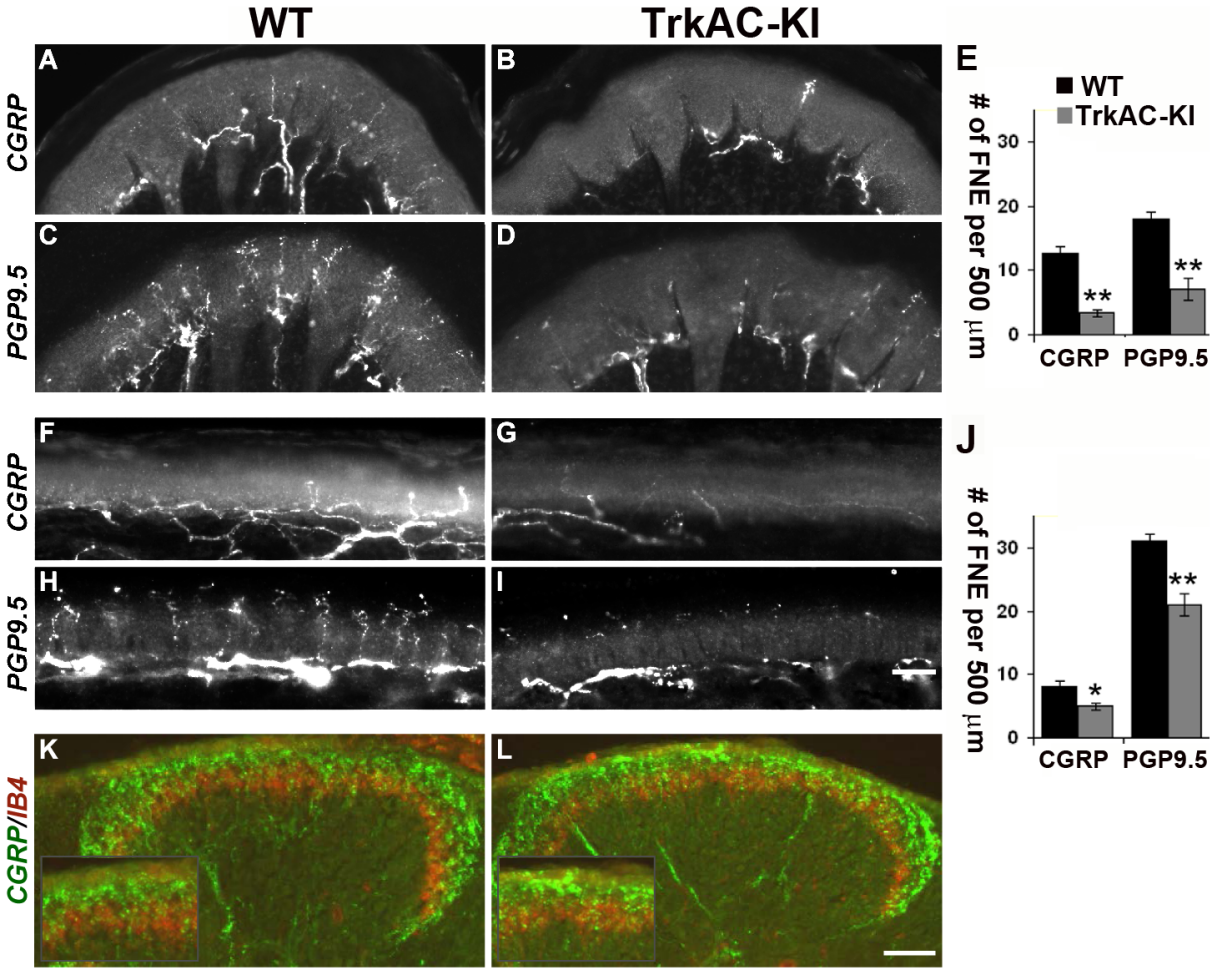
Peripheral, but not central innervation is drastically reduced in TrkAC-KI mice. (A–D) Peptidergic (CGRP-positive) and total (PGP9.5-positive) fiber innervation is decreased in thick glabrous skin of adult *TrkAC-KI* hindlimbs. (E) Free nerve endings (FNE) counts in thick glabrous skin. Shown are means ± s.e.m. from 8–10 sections from two animals per genotype (* p>0.05, ** p<0.01). (F–I) Peptidergic (CGRP-positive) and total (PGP9.5-positive) fiber innervation is decreased in thin glabrous skin of adult *TrkAC-KI* hindlimbs. (J) Free nerve ending (FNE) counts in thin glabrous skin. Shown are means ± s.e.m. from 8–10 sections from two animals per genotype (* p>0.05, ** p<0.01). (K,L) Central projections are normal in *TrkAC-KI* mice. Peptidergic (CGRP-positive, green) and nonpeptidergic(IB4-positive, red) fibers normally innervate adult spinal cord in *TrkAC-KI* mice. Scale bar is 50 µm.

Anatomically, peptidergic and nonpeptidergic nociceptors innervate distinct peripheral and central targets in the body. In the periphery, nonpeptidergic fibers mainly innervate the skin, whereas peptidergic fibers project to most parts of the body in addition to skin. Moreover, in the glabrous skin, they innervated distinct layers of epidermis. Centrally, peptidergic and nonpeptidergic fibers terminate in distinct lamina in the dorsal horn of the spinal cord [18]. Since NGF/TrkA signalling is critical for peripheral target innervation [4], [8], we then questioned whether expression of TrkAC chimeric protein in nociceptors would have an effect on axonal extension *in vivo*. Surprisingly, despite normal survival and maturation of nociceptors described above, we observed a drastic decrease in peptidergic (CGRP-positive) fibers both in thin (Figures 3A, 3B and 3E) and thick (Figures 3F, 3G, and 3J) glabrous skin of adult *TrkAC-KI* mice comparing to wild type littermate controls. Since the number of CGRP-positive DRG neurons is normal in these mice (Figures 2A and 2B), the observed innervation deficit is due to target innervation defect and not due to cell death. Nonpeptidergic neurons depend on NGF/TrkA signalling during early development and thus could also be affected in *TrkAC-KI* mice. We therefore used pan-axonal marker PGP9.5 on skin sections from *TrkAC-KI* and control mice. Indeed, the reduction in PGP9.5-positive fibers in thin glabrous skin from *TrkAC-KI* mice was even greater than reduction in CGRP fibers, suggesting that target innervation by nonpeptidergic neurons was also affected (Figure 3J). The peripheral innervation defect was also observed in hairy skin, where both CGRP and PGP9.5 positive fibers appeared disorganized and drastically reduced in numbers (Figures S5A–D).

Consistent with the observed skin innervation defect, sensory neurons from *TrkAC-KI* mice also showed abnormal NGF-dependent axonal extension *in vitro*. When exposed to NGF, embryonic DRG explants from *TrkAC-KI* mice grew out neurites at lower density comparing to DRG explants from wild type control embryos (Figures S6A–C). Neurites from mutant explants also appeared more tortuous comparing to neurites from wild type explants. However, this is most likely a consequence of difference in outgrowth density. A similar neurite outgrowth deficit was observed in cultured dissociated DRG neurons grown in presence of NGF. There were a significantly lower number of neurite-bearing cells in cultures from *TrkAC-KI* embryonic DRGs comparing to wild type dissociated neuron cultures (Figures S6D–F). In order to make sure that this difference was not due to differences in neuronal viability, the dissociated neurons were always labelled with anti-caspase-3 antibody. Only caspase-3 negative neurons were counted. Interestingly, the length and morphology of those *TrkAC-KI* neurons that did grow neurites were similar to that of wild type (Figure S6G), suggesting that TrkAC-expressing neurons are capable growing axons and that the basal axonogenesis process is intact in these cells.

Interestingly, both peptidergic (CGRP-positive, green) and nonpeptidergic (IB4-positive, red) fibers innervated adult spinal cord normally in *TrkAC-KI* mice (Figures 3K and 3L). This is not surprising, since DRG neurons projected to spinal cord even in Bax-deficient mice lacking NGF/TrkA signaling [4]. Thus, despite grossly normal survival and maturation of nociceptors in *TrkAC-KI* mice, innervation of adult skin by these neurons is specifically disrupted.

### Abnormal adult skin innervation in *TrkAC-KI* mice is due to a developmental defect

**Figure 4:**
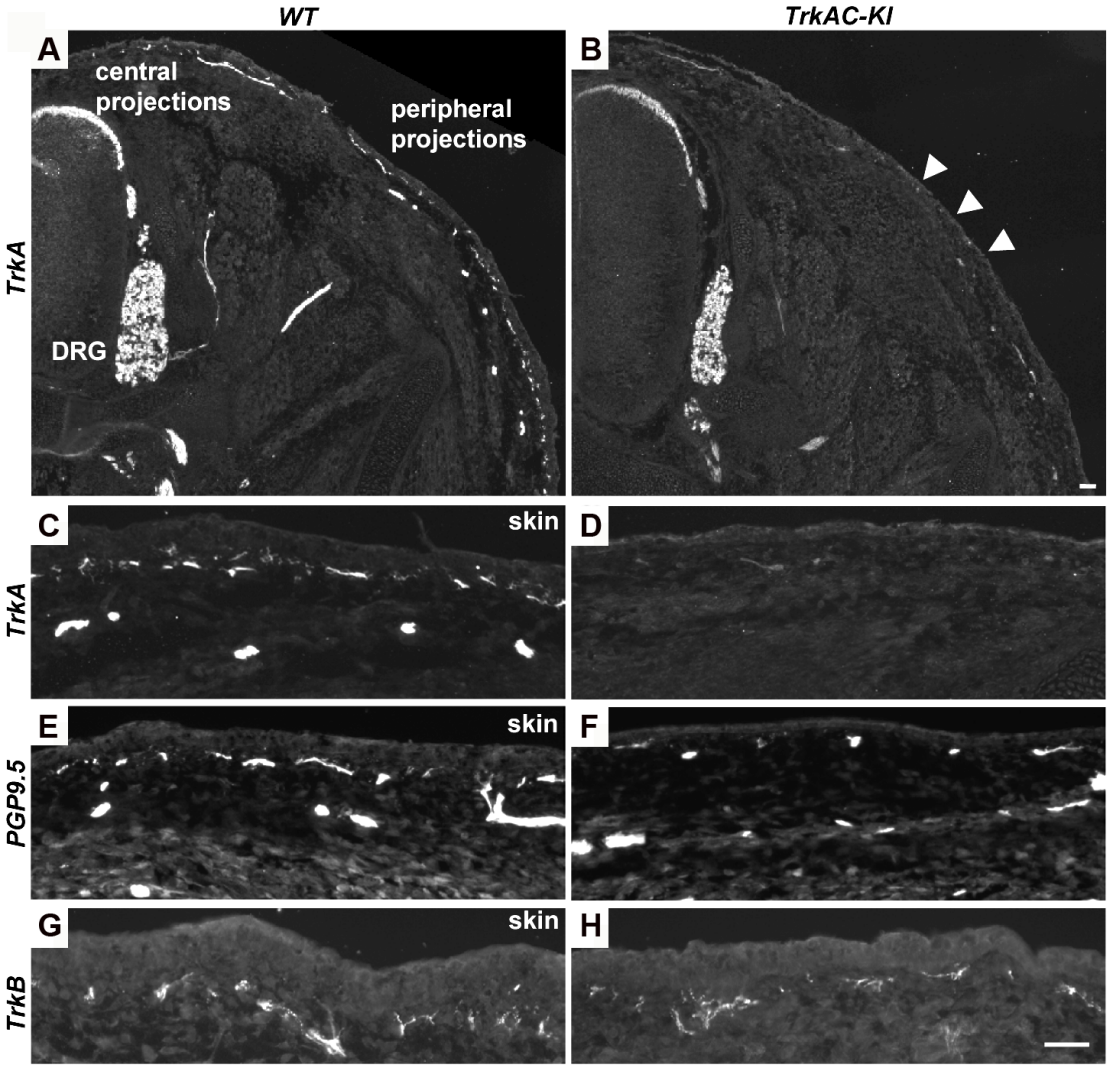
Peripheral innervation defect is evident during embryonic development. (A–D) Sections of E14.5 *TrkAC-KI* and control embryos stained with anti-TrkA antibody recognizing both TrkA and TrkAC proteins. Labeling of DRGs and projections to the spinal cord (central projections) is similar in both mutant and control animals while epidermal innervation is greatly decreased. (E and F) There are less PGP9.5 positive fibers in the skin of *TrkAC-KI* comparing to control embryos. (G and H) Skin innervation by TrkB-positive fibers is not changed in *TrkAC-KI* embryos. Scale bar is 50 µm.

We then investigated whether the reduced peripheral innervation in adult skin was due to a developmental defect or due to loss of axons at later stages. Immunofluorescent staining with anti-TrkA antibody showed robust labeling of DRGs and central projections in both *TrkAC-KI* and control E14.5 embryos, while drastic decrease in skin innervation was evident in *TrkAC-KI* comparing to control embryos (Figures 4A–D). Reduced skin innervation was also observed when stained with anti-PGP9.5 antibody, suggesting that the reduction in TrkA-positive fibers is due to defects in innervation and not due to lack of TrkA protein reactivity in axons (Figures 4E and 4F). Notably, skin innervation by mechanosensory TrkB positive fibers, known to innervate specialized structure in the dermis, was normal in *TrkAC-KI* embryos at this developmental stage (Figures 4G and 4H). Thus, even though TrkAC chimeric receptor supported survival, correct molecular maturation and normal central innervation of former TrkA-expressing neurons, it was not able to promote normal peripheral target innervation by these neurons during development.

### Expression of multiple axonal outgrowth molecules is affected in *TrkAC-KI* DRGs

**Figure 5:**
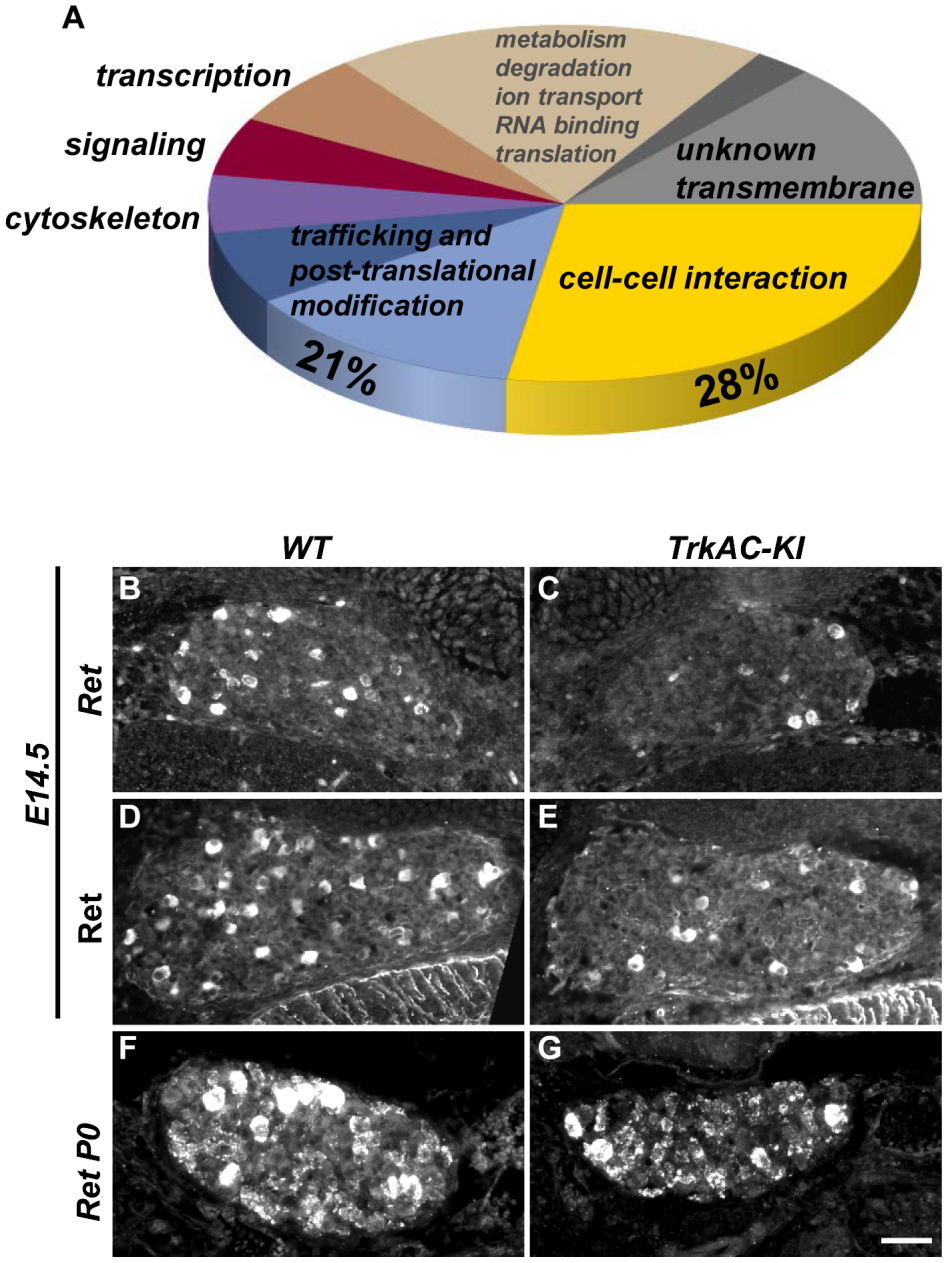
Microarray screen for differentially expressed genes in DRGs from E14.5 *TrkAC-KI* embryos reveal a number of genes potentially responsible for NGF-dependent axonal growth. (A) Results of microarray experiment comparing gene expression between DRGs from *TrkAC-KI* and control E14.5 embryos. 28% of identified genes encoded for cell-cell interaction and cell adhesion molecules, and 21% for trafficking and post-translational modification proteins. Non-coding genes, as well as genes with both up- and down-regulated probes, were excluded from the data set presented in this figure (123 genes). Ret is downregulated 2 fold in our microarray. (B–G) Ret expression in small neurons is delayed *in TrkAC-KI* mice, as shown by *in situ* hybridization (B,C) and antibody labeling of E14.5 DRGs (D,E) and *in situ* hybridization of P0 DRGs (F,G) from mutant and control animals. Scale bar is 50 µm.

Previous attempts to identify NGF-responsive genes yielded a large data set obtained from microarray experiments on neurotrophin mutants with defects in multiple NGF-dependent processes [19], [20]. In *TrkAC-KI* mice, however, the axonal growth deficit is accompanied by grossly normal survival and phenotypic differentiation of sensory neurons. Therefore, *TrkAC-KI* mice present a unique opportunity to identify genes specifically responsible for NGF-dependent axonal outgrowth without the confounding presence of genes controlling NGF-dependent survival and maturation. We seized this opportunity and performed a genome-wide screen comparing gene expression in DRGs from E14.5 *TrkAC-KI* and control embryos. A total number of 140 genes was identified by our microarray screen using FDR (False Discovery Rate) [21] cutoff 0.01 and fold-change of >1.5 (Table S1). Of interest, the 5 probes showing the highest downregulation in our screen corresponded to the endogenous *TrkA*-specific probes, expression of which is completely abolished in *TrkAC-KI* mice (Table S1 and Figure 1B). Remarkably, almost a third of genes (28%) that were up- or downregulated in *TrkAC-KI* DRGs encoded for proteins involved in cell-cell interaction and cell adhesion, processes critical for axonal pathfinding and growth (Figure 5A). Second largest (21%) group of genes encoded for proteins that are likely to play a role in trafficking and post-translational modification (Figure 5A). Dysfunction of the fine-tuned trafficking machinery needed to deliver building blocks to the extending tip of a growing axon could indeed be in part responsible for the peripheral innervation defect observed in *TrkAC-KI* mice. Interestingly, 16 out of 19 unknown genes identified in our screen encoded for transmembrane proteins, suggesting that they might also participate in these processes. A number of genes deregulated in *TrkAC-KI* mice have been previously identified in microarray screens for NGF-dependent genes, such as Cntn1, Cntn4, Mapk8, Rgs4 and Ret [19], [20]. Interestingly, while Ret expression was normal in DRGs from P0 and adult *TrkAC-KI* mice (Figures 5F, 5G and 2C, 2D), both protein and mRNA levels of Ret were decreased in small-diameter neurons at E14.5 (Figures 5B–E). The expression of Ret in large-diameter neurons, representing the “early Ret” population of rapidly adapting mechanoreceptors which do not express TrkA [22], remained unchanged (Figures S4Q–V). Ret is necessary for establishment and maintenance of epidermal innervation by nonpeptidergic sensory neurons [5], [22]. Thus, delayed expression of Ret or downregulation of other NGF-dependent axonal growth genes identified by our microarray screen could in part contribute to the target innervation phenotype observed in *TrkAC-KI* mice.

### Detection of temperature is moderately affected in *TrkAC-KI* mice

**Figure 6:**
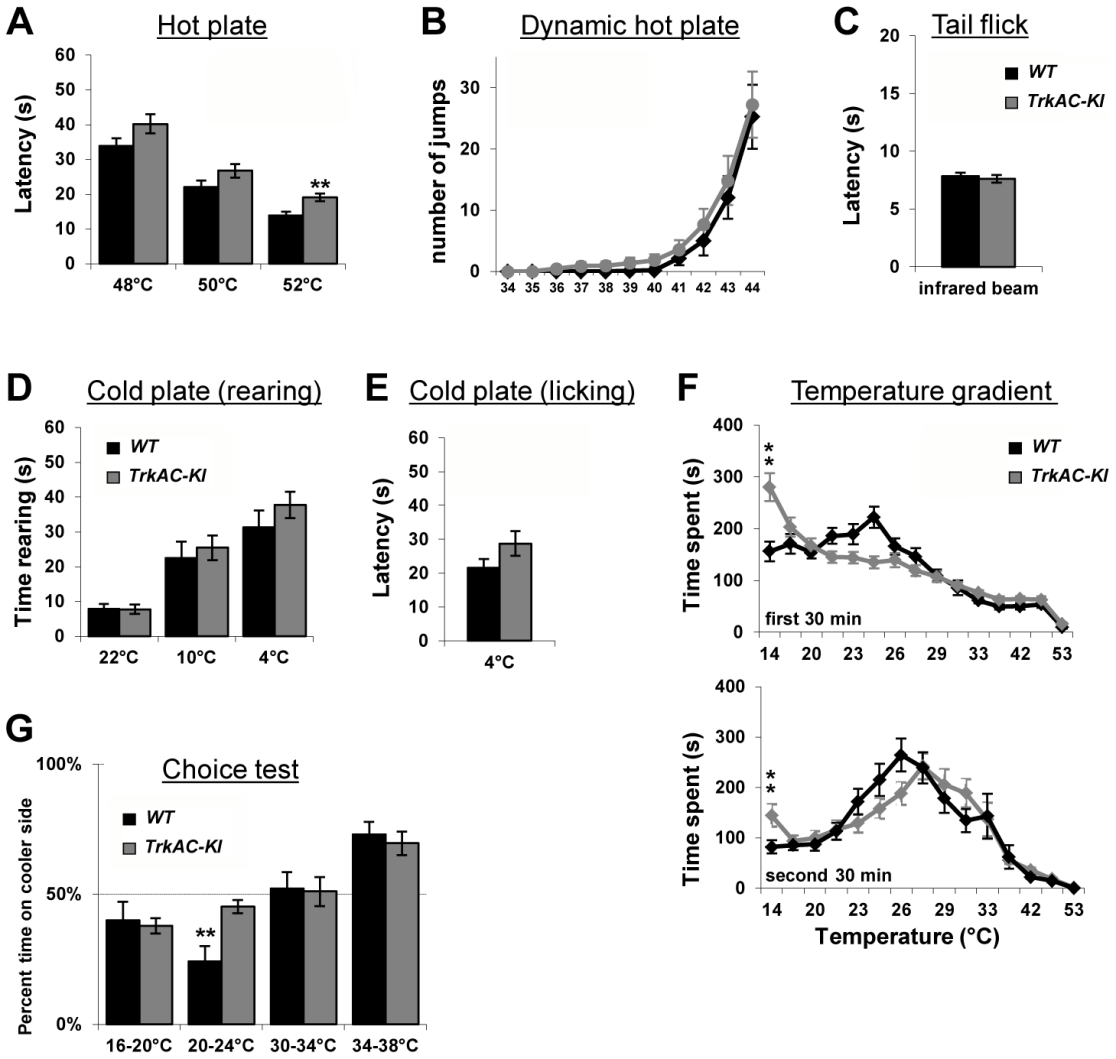
Abnormal temperature sensitivity in *TrkAC-KI* mice. (A) *TrkAC-KI* mice showed increased latency of response at 52°C, but not at 48°C or 50°C (n = 9 for wild type and 11 for *TrkAC-KI*). (B–E) There was no difference between control and *TrkAC-KI* mice in the Dynamic Hot Plate (n = 14 for wild type and 16 for *TrkAC-KI*), Tail flick (n = 10 for wild type and 13 for *TrkAC-KI*), Cold Plate/Rearing (n = 10 for wild type and 8 for *TrkAC-KI*), or Cold Plate/Licking (n = 10 for wild type and 13 for *TrkAC-KI*) tests. (F) *TrkAC-KI* mice did not avoid cooler (14°C) area of the Temperature Gradient, suggesting that they were less sensitive to cool temperatures (n = 24 for wild type and 26 for *TrkAC-KI*). (G) *TrkAC-KI* mice spend significantly more time on cooler side during 20–24°C choice test (n = 11 for wild type and 15 for *TrkAC-KI*), while behaving similarly to control mice in 16–20°C (n = 11 for wild type and 15 for *TrkAC-KI*), 30–34°C (n = 11 for wild type and 16 for *TrkAC-KI*) or 34–38°C (n = 11 for wild type and 16 for *TrkAC-KI*) choice tests. Wild type: black bars, *TrkAC-KI*: gray bars. Data represent mean ± s.e.m. * p<0.05, ** p<0.01.

**Figure 7:**
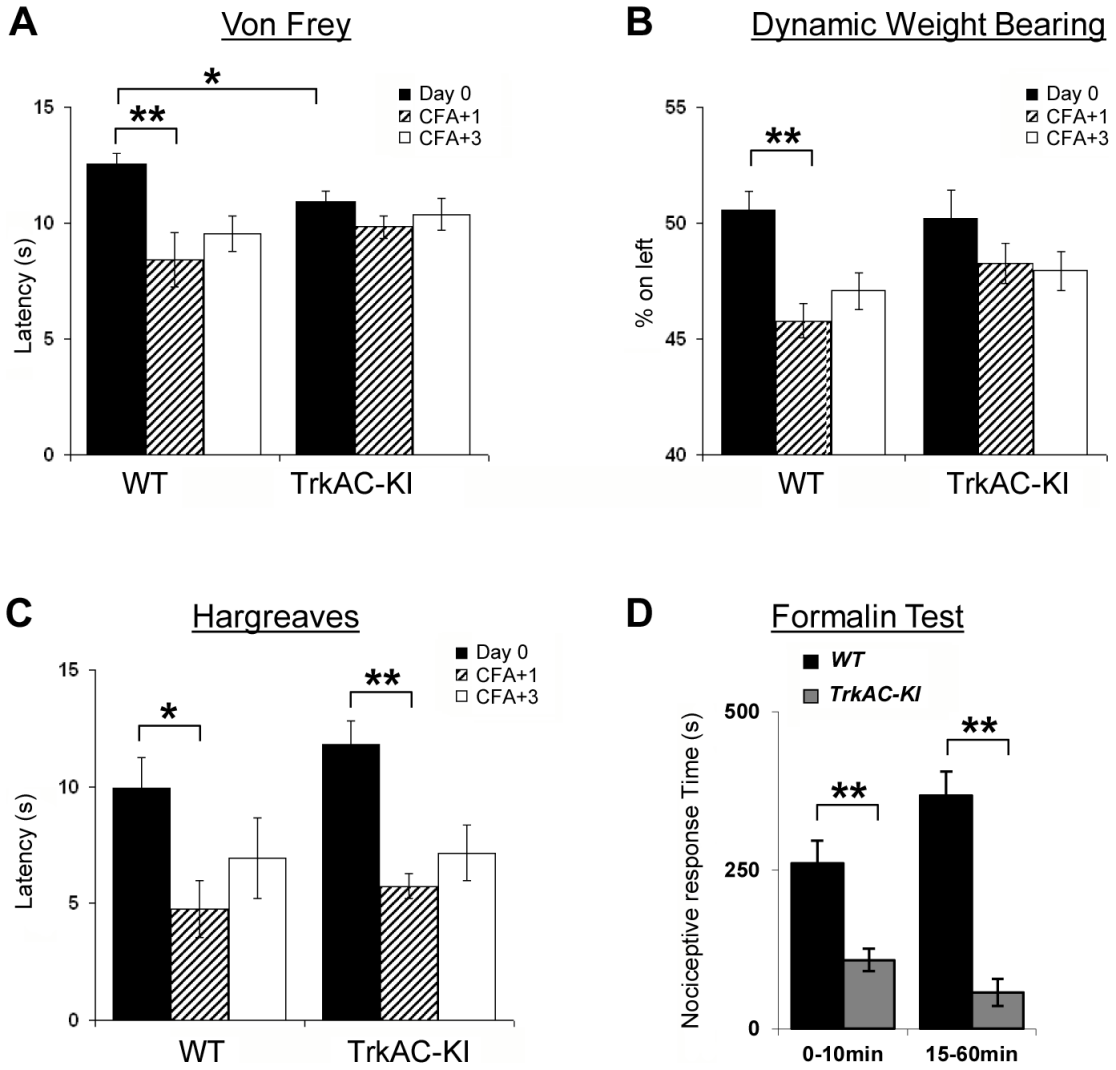
Abnormal mechanical and chemical pain response in *TrkAC-KI* mice. (A) Latency to mechanical stimulation using Von Frey apparatus was significantly lower in control mice one day after CFA injection, while *TrkAC-KI* mice did not show this response. Of note, the baseline latency to mechanical stimulation was lower in mutant mice (n = 9 for wild type and 13 for *TrkAC-KI*). (B) Lack of mechanical hypersensitivity after inflammation was also evident from a Dynamic Weight Bearing test. For *TrkAC-KI* mice, the weight distribution between inflamed and non-inflamed hindpaws was equal one day after CFA injection, while control mice favored the non-injected paw (n = 10 for wild type and 9 for *TrkAC-KI*). (C) Both *TrkAC-KI* and control mice developed thermal hyperalgesia one day after CFA injection (n = 8 for wild type and 6 for *TrkAC-KI*). The CFA effect (difference in latency between Day0 and day CFA+1) was significantly different between *TrkAC-KI* and wild type mice for Von Frey and DWB tests (A and B), but not for Hargreaves test (C). (D) *TrkAC-KI* mice exhibited severe deficit in pain from chemical injury when tested for nociceptive response after intraplantar injection of 10 µl of 2% formalin. Comparing to wild type littermates, *TrkAC-KI* mice had drastically reduced time of hindpaw shaking and biting during the first (0–10 min) and second (15–60 min) pain phases (n = 7 for each genotype). Data represent mean ± s.e.m * p<0.05, ** p<0.01.

NGF/TrkA signaling is critical for development of nociceptive neurons which detect a variety of stimuli. However, behavioural analyses of mice mutant for NGF, TrkA, as well as NGF/Bax and TrkA/Bax double knockout mice have not been possible, since these animals die shortly after birth [1], [2], [4]. Unlike these mouse mutants, *TrkAC-KI* mice survived until adulthood and behaved normally in general locomotion and anxiety tests (Figures S7A and S7B), thus for the first time allowing behavioral analysis of mice with genetically altered NGF/TrkA signaling. We therefore subjected *TrkAC-KI* mice to a large battery of somatosensory tests. When tested on a hot plate at three different noxious temperatures, *TrkAC-KI* mice showed a decreased response at 52°C (Figure 6A), whereas heat threshold responses measured by the dynamic hot plate (Figure 6B), tail flick (Figure 6C) and Hargreaves protocols (Figure 7D) were not affected in *TrkAC-KI* mice. Since the number of TRPM8-expressing neurons is significantly decreased in *TrkAC-KI* mice, we tested the ability of these mice to respond to cold temperature. While rearing behavior and licking response latencies to cold stimuli were comparable between *TrkAC-KI* and control mice (Figures 6D and 6E), we observed a greater tolerance to cold (14°C) temperature during the thermal gradient protocol in *TrkAC-KI* compared to control mice. In this test, mice were allowed to move freely in a corridor with temperature gradient spanning from 14°C to 53°C for 1 hour. While attracted to the corner regions of the testing area, mice are generally repelled by the two extreme temperatures present in these parts. *TrkAC-KI* mice, however, spend a significant amount of time in the 14°C corner area during both periods: the exploration period (first 30 minutes) and the established preferred temperature period (second 30 minutes) (Figure 6F). To further examine this thermal selection phenotype, control and *TrkAC-KI* mice were subjected to series of two-temperature choice assays. In line with the decreased sensitivity to cold temperature observed in the gradient test, when given a choice between 20°C and 24°C, *TrkAC-KI* mice showed no preference for either side, whereas control mice displayed strong preference for the warmer area (Figure 6G). Mice of both genotypes behaved similarly when presented with other tested temperature choices (16°–20°, 30°C–34°C and 34°C–38°C) (Figure 6G). Together, these data showed that, despite drastic defects in skin innervation, temperature sensitivity is only moderately affected in *TrkAC-KI* mice.

### *TrkAC-KI* mice display severe deficit in response to noxious mechanical and chemical stimuli

In the last decade, growing evidence has implicated NGF/TrkA signalling as a major component contributing to many persistent pain states, especially those associated with inflammation [23]. We thus tested *TrkAC-KI* mice for ability to develop an inflammatory pain response to complete Freund's adjuvant (CFA) intraplantar injections, using automated Von Frey apparatus. One and three days after injections, CFA-induced mechanical hyperalgesia was highly pronounced in control but not in *TrkAC-KI* mice, when compared to the response before CFA injection (Figure 7A). However, since the latency of acute response to Von Frey stimulus before injection (Day 0) was lower in *TrkAC-KI* mice (Figure 7A), we also used another mechanical stimulation test, a newly engineered automated Dynamic Weight Bearing (DWB) device. This apparatus uses pressure captors that allow measuring the weight bore on paws of a freely moving mouse. In this test, mice are not acclimatized to the testing apparatus in order to maximize exploration behaviors. Before CFA-induced inflammation (day 0), the percentage of mouse weight distributed on both ipsilateral and contralateral hindpaws was equivalent between the two genotypes (Figure 7B). At one and three days post inflammation, control mice showed a marked disequilibrium towards the contralateral paw (the non-inflamed paw), while the hindpaw weight distribution of the *TrkAC-KI* mice was unchanged (Figure 7B). Surprisingly, post-inflammatory response to thermal stimulation was normal in *TrkAC-KI* mice (Figure 7C). In all experiments, paw swelling after CFA injection was comparable between *TrkAC-KI* and control mice. Together, the Von Frey and the DWB tests demonstrate that mechanical hypersensitivity response to tissue injury is disrupted in *TrkAC-KI* mice. To evaluate chemical sensitivity of *TrkAC-KI* mice, we opted for the formalin test. This test is a tonic model of continuous pain resulting from formalin-induced tissue injury. In rodents, intraplantar injection of formalin triggers a biphasic pain-like response characterized by flinching, licking and biting behaviors. It is generally admitted that the first phase results from activation of nociceptors at the site of injection while the second phase is largely due to central sensitization of spinal cord circuits as well as due to peripheral inflammation [24]. Injection of 10 µl of 2% formalin triggered robust first and second pain responses in the control mice, while these two behaviors were drastically reduced in *TrkAC-KI* mice, including an almost complete suppression of the second pain response (Figure 7D). These data demonstrate that tissue injury-induced chemical hypersensitivity is severely impaired in *TrkAC-KI* mice and highlight the importance of NFG/TrkA signaling in the development and function of nociceptive neurons.

## Discussion

Neurotrophins control a number of different aspects of sensory neuron development [25]. Given its biological importance, numerous studies attempted to dissect the precise mechanisms of NGF/TrkA signaling, mostly by *in vitro* approaches using cultured neurons or neuron-like cell lines. However, our understanding of how this signaling affects the development of sensory neurons remains limited due to the challenging nature of experiments aimed at modulating Trk signaling *in vivo*. One study has previously addressed this issue by replacing the endogenous TrkA by TrkC using a knock in approach [9]. A subset of nociceptors in these mice developed into proprioceptors, which could be explained either by an instructive role of intracellular TrkC signaling or by a switch in responsiveness to an extracellular factor. We now report a novel mouse mutant expressing a chimeric TrkA/TrkC receptor in which the nociceptors respond to NGF but activate the intracellular signaling through TrkC intracellular domain. These mice show specific developmental target innervation defects in otherwise grossly normal nociceptive neurons. The fact that TrkAC-expressing sensory neurons retain their nociceptive fate argues against the hypothesis that activation of intracellular TrkC signaling can set in motion proprioceptor-specific developmental programs. Our results, therefore, highlight the critical role of extrinsic target derived factors in determining the fate of sensory neurons. It is likely that these factors are encountered by a growing sensory axon before it reaches its final destination, since TrkAC-positive nociceptors express most of the appropriate molecular markers even though they do not project to epidermis correctly.

Even though intracellular domains of TrkA and TrkC receptors share significant amino acid similarity, several differences in activation of downstream effectors as well as binding of interacting proteins have been reported [12], [26]–[28]. Remarkably, despite these differences, we show for the first time that the intracellular parts of these two receptors are interchangeable *in vivo* for supporting nociceptor survival and maturation.

It is well established that NGF/TrkA-dependent signaling controls nociceptors survival, specific marker expression and peripheral target innervation [1], [2], [4]. How can the same ligand/receptor complex activate downstream pathways controlling such distinct developmental outcomes? One hypothesis is that NGF-dependent signals instructing target innervation differ, either qualitatively or quantitatively, from signals controlling nociceptor survival and maturation. Currently available mouse mutants for neurotrophins and their receptors do not allow testing this hypothesis as these animals have deficits in multiple aspects of development [1]–[4]. We now show that survival and phenotypic maturation of nociceptors can be uncoupled from axonal growth by altering the intracellular part of the NGF receptor TrkA. What are the molecular mechanisms leading to uncoupling of these developmental processes in *TrkAC-KI* mice? One possibility is that the amount of NGF-activated signaling necessary for target innervation is higher than that required for supporting neuronal survival and expression of nociceptive markers. Indeed, our data show that replacing the intracellular part of TrkA with that of TrkC activated proteolytic processes leading to lower amount of the mature form of TrkAC receptor in mutant embryos. It has been recently shown DRG explant neurites are more responsive to an NGF gradient than to an absolute NGF concentration [29]. It is possible that interpretation of this gradient is defective in TrkAC-expressing neurons. Another explanation is that target innervation by nociceptors is controlled by TrkA-specific downstream transduction pathways, which could be specifically disrupted in *TrkAC-KI* mice. Given structural similarity of TrkA and TrkC, the vast majority of intracellular effector proteins interact with either receptor [25]. There are few proteins, however, that bind differentially to the intracellular domains of these two receptors, such as GIPC1 [27], Grit [28] and Nedd4L [26], possibly leading to activation or modulation of distinct downstream pathways. A recent study has also revealed fundamental differences between TrkA, TrkB and TrkC in instructing neuronal death both *in vitro* and *in vivo* [30]. Moreover, introducing a Sch site mutation in TrkB and TrkC receptors *in vivo* had distinct effects on vestibular and cochlear neurons respectively [31]. Finally, structural differences between TrkA and TrkAC receptors could lead to different activation levels of downstream effectors. Indeed, previous *in vitro* studies demonstrated that while both TrkA and TrkC receptors activated ERK and Akt pathways, they did so to a different extent, leading to distinct effects on axonal morphology [12]. Accordingly, our results on cultured sensory neurons from mutant and wild type embryos clearly show that the chimeric TrkAC receptor has different signaling properties comparing to that of TrkA, since lower amount of this receptor is able to activate wild type levels of pERK and pAkt. Regardless of the molecular mechanism, *TrkAC-KI* mice demonstrate for the first time that certain developmental processes, such as nociceptor survival and maturation, are less sensitive to qualitative and quantitative changes in Trk signaling, while other aspects of development, such as peripheral target innervation, might require precise levels of activation of specific pathways.

Both in humans and mice, loss-of-function mutations affecting NGF/TrkA signalling lead to marked insensitivity to pain [1], [2], [32] and sequestering NGF or TrkA has analgesic effects under various experimental conditions [23], [33]. However, the early lethality of NGF and TrkA mutant mice greatly impeded our understanding of the consequences of altered NGF/TrkA signalling in pain processing during postnatal stages. Our behavioural experiments revealed that *TrkAC-KI* mice have severe deficits in response to injury-induced mechanical and chemical pain. What are the mechanisms responsible for this phenotype? We favor the hypothesis that peripheral inflammation or chemical injury stimuli are not reaching the appropriate sensory fibers to generate an adequate response because of the drastic defects in skin innervation present in *TrkAC-KI* mice. In adult mice, injury-induced mechanical and chemical hypersensitivity require an intricate cross talk between peptidergic TrkA^+^ and nonpeptidergic Ret^+^ nociceptors. Indeed, genetic ablation of Nav1.8-expressing neuronal population, which included nearly all nonpeptidergic and a large fraction of peptidergic neurons, caused severe mechanical and chemical (formalin) hyposensitivity in mice [34]. Thus, it is most likely that the loss of injury-induced mechanical hypersensitivity and the decrease in response to formalin in *TrkAC-KI* mice is due to a dual defect: the developmental decrease in skin innervation by all nociceptors and the decreased amount of the chimeric TrkAC receptor in adult peptidergic neurons. Alternatively, the pain phenotypes could be caused by rewiring of the neuronal circuits in the spinal cord, present at the synaptic level despite the apparently normal central innervation morphology observed in *TrkAC-KI* mice. A combination of electrophysiological, behavioural and molecular studies of *TrkAC-KI* mice will distinguish between these possibilities and will greatly contribute to improving our understanding of the molecular mechanisms of nociception and pain.

It has been recently shown that acute deletion of Ret in adult mice leads to almost complete retraction of nonpeptidergic fibers from epidermis within two weeks [22], suggesting that nociceptive innervation of mammalian skin might be much more dynamic than previously thought. This plasticity in innervation is likely to play a significant role in pain perception in both normal and pathological states. Indeed, subcutaneous perfusion with NGF-sequestering molecules induced hyposensitivity to noxious thermal stimuli only after 5 days, with a similar latent period for recovery of sensitivity after the perfusion was stopped [35]. Retraction or reorganization of nociceptive axonal arbors could be responsible for this delayed effect. Major pain deficits in *TrkAC-KI* mice exhibiting drastic reduction in skin innervation are consistent with this hypothesis. NGF, a key player in the establishment of peripheral target innervation during embryonic development, has also been shown to alter the axonal arborisation in the adult skin [36]. Thus, it is likely that molecular mechanisms that govern the extension of embryonic sensory axons during development could also play a role in dynamics and plasticity of adult epidermal innervation. *TrkAC-KI* mice, therefore, represent an excellent model to study these processes, potentially leading to development of novel pain-controlling therapies. Finally, this mouse model also represents an invaluable tool to address the functions of other populations of neurons that respond to NGF/TrkA signalling such as sympathetic and cholinergic forebrain neurons.

## Materials and Methods

### Ethics Statement

The animal experiments were approved by the Comit? d ?thique de Marseille (reference number 14-08112010).

### Generation of *TrkA^TrkAC^* (*TrkAC-KI*) knock-in mice

See Protocol S1 for details.

### Western blot on DRG extracts

For each genotype, DRGs were dissected from individual E14.5 embryos as well as from adult animals and lysed in the buffer containing 150 mM NaCl, 10 mM Tris PH7.4, 1 mM EDTA, 10 mM NaF, 1 mM Vanadine, 0.25% Na Deoxycholate, 1% NP40 and protease inhibitors. In order to quantify the amount of TrkA and TrkAC protein in embryonic DRGs, for each independent experiment (four total), lysates from individual control, heterozygous and *TrkAC-KI* embryos were run in parallel. Western blots were probed with anti-TrkA (generous gift from Dr. L. Reichardt, University of California San Francisco), recognizing both TrkA and TrkAC proteins, as well as anti-ERK (Cell Signaling, #9102), followed by revelation using ECL Plus kit (GE Healthcare Amersham) and MS Kodak film. Films were then scanned, making sure that the bands were not oversaturated. Intensities of TrkA or TrkAC bands, as well as corresponding loading control ERK bands were then measured using ImageJ program. After normalizing the intensity values of TrkA and TrkAC bands using the values obtained for ERK bands to control for loading differences, TrkAC amounts in heterozygous and homozygous embryos were quantified as percentage of TrkA in wild type embryos.

### DRG explants and cultures of dissociated DRG neurons

For explant culturing, DRGs were dissected from E13.5 embryos obtained from a heterozygote cross, deposited on coverslips coated with poly-D-lysine(50 µg/ml, Sigma, P7405) and laminin (10 µg/ml, Sigma, L2020) and grown overnight in Neurobasal media (Invitrogen) supplemented with 2% B27 (Invitrogen), 2 mM glutamine and 1 mM Na pyruvate, in presence of 50 ng/ml NGF (Alomone Labs, N-240, lot # NF-11). DRG explants were then labeled with mouse anti-neurofilament antibody (NF (160 kD) NN18, Sigma, N5264) and photographed at 2.5× magnification. NIH ImageJ program was used to measure the neurite density in the area defined by two concentric circles 100 µm and 200 µm away from the explant border.

Dissociated DRG cultures were prepared following standard protocols. Briefly, DRG from E14.5 embryos were collected in cold DMEM. After trypsinization (10 min at 37°C), tissue was triturated using two fire-polished Pasteur pipettes and washed in Neurobasal media. Cells were then plated at low density (50 000–200 000 cells per well in a 4-well plate) on coverslips coated with poly-D-lysine/laminin, grown overnight (18 hours) in Neurobasal media containing 50 ng/ml NGF and 10 µM mix of FDU/U (5-Fluoro-2′-deoxyuridine, Sigma, F0503 and Uridine, Sigma, U3003) in order to prevent growth of non-neuronal cells. DRG neurons were then stained with mouse anti-neurofilament (Sigma) and rabbit anti-caspase3 (Cell Signaling) antibodies. In order to avoid bias in random field selection, an area of 25 fields (5×5) in the center of the coverslip was imaged at 10× magnification using MozaicX option on Apotome Z1. This area, corresponding to approximately 12 µm^2^, was divided into 12 1 µm^2^ areas in Photoshop. Neurons with and without neurites were identified by neurofilament staining and morphology. The length of neurites was measured using NeuronJ plug-in for NIH ImageJ program.

For cultures used in NGF-stimulation experiments, neurons were first grown overnight in presence of 50 ng/ml NGF and FDU/U, then washed twice in NGF-free Neurobasal medium and starved for 48 hours in NGF-free Neurobasal medium containing 2 µM caspase inhibitor Boc-D(Ome)-FMK (Biovision, #1160). DRG neurons were then stimulated with 100 ng/ml NGF for 5 and 15 minutes, by gently replacing half of the medium present in a well with equal amount of medium containing 200 ng/ml NGF. For non-stimulated cultures, half of the medium was replaced by an equal amount of NGF-free medium for 5 minutes. After stimulation, plates were immediately placed on ice, washed with ice-cold PBS and lysed in buffer containing 150 mM NaCl, 10 mM Tris PH7.4, 1 mM EDTA, 10 mM NaF, 1 mM Vanadine, 0.25% Na Deoxycholate, 1% NP40 and protease inhibitors. The following antibodies were used for Western blotting: anti-TrkA (generous gift from Dr. L. Reichardt, University of California San Francisco), anti-pErk1/2 (Cell Signaling, #9106), anti-pAkt (Cell Signaling, #9271S), anti-ERK (Cell Signaling, #9102).

### *In situ* hybridization and immunofluorescence

*In situ* hybridization and immunofluorescence were carried out following standard protocols [9]. To obtain adult tissues, animals were deeply anesthetized with a mix of ketamine/xylazine and then transcardially perfused with an ice-cold solution of paraformaldehyde 4% in PBS (PAF). After dissection, DRGs were post-fixed for at least 24 hours in the same fixative at 4°C. Embryos were collected in ice-cold PBS, and fixed for 24 h in 4% PAF. Adult back skin and footpads were excised from deeply anesthetized animals and incubated in 5% formaldehyde, 15% v/v picric acid in PBS overnight at 4°C. Tissues were then transferred into a 30% (w/v) sucrose solution for cryoprotection before being frozen and stored at −80°C. Samples were sectioned at 10–30 µm using a standard cryostat (Leica).

RNA probes were synthesized using gene-specific PCR primers and cDNA templates from embryonic or adult mouse DRG. Double fluorescent *in situ* hybridization was carried out using a combination of digoxigenin and fluorescein/biotin labeled probes. Probes were hybridized overnight at 55°C and the slides incubated with the horseradish peroxidase anti-digoxigenin/fluorescein/biotin antibody (Roche). Final detection was achieved using fluorescein/cy3/cy5 TSA plus kit (Perkin Elmer). For double fluorescent *in situ* experiments, the first antibody was inactivated using H_2_O_2_ treatment.

For *in situ* probes, the following nucleotide positions were used. TrkA-Cterm, 2232–2573 of NM_001033124; TrkC-Cterm, 1360–1881 of NM_008746; PV, 80–594 of NM_013645; TrpM8, 1410–1980 of AF481480; TrpA1, 617–1518 of NM_177781, TrpV1, 1521–2065 of NM_031982; MrgB4, 1–860 of NM_205795; TrpC3, 2599–3514 of NM_019510; GFRalpha1, 1746–2913 of NM_010279; GFRalpha2, 2520–3287 of NM_008115; GFRalpha3, 1318–1952 of NM_010280; MrgA1, 89–917 of NM_153095; Ret, 1370–1845 of NM_009050.

For immunofluorescence, primary antibodies were diluted in PBS-10% donkey serum (Sigma)-3% bovine albumin (Sigma)-0.4% triton-X100 and incubated overnight at 4°C. Corresponding donkey anti-rabbit or anti-goat Alexa 488 or 555 (Molecular Probes) were used for secondary detection. Primary antibodies used in this study are as follows: rabbit anti-TrkA 1∶2000 (generous gift from Dr. L. Reichardt, University of California San Francisco), rabbit anti-Runx1 1∶4000 (generous gift from Dr. T. Jessell, New York University School of Medicine), goat anti-TrkC 1∶500 (R&D systems), goat anti-Ret 1∶500 (R&D systems) and rabbit anti-CGRP 1∶2000 (Chemicon).

### Microarray analysis

Spinal columns from E14 embryos from multiple litters were quickly dissected in Hank's Buffered Salt Solution (HBSS) and placed in RNAlater (Ambion) overnight at 4°C awaiting genotyping. DRGs were then dissected out and homogenized using glass beads in Precellys 24 (Bertin Technologies, France), followed by RNA extraction using RNeasy mini kit (Qiagen). RNA was then concentrated by precipitation with lithium chloride (2.5 M final concentration). The amount and quality of RNA was assessed using NanoDrop (NanoDrop Technologies) and Experion system (BIO-RAD). Three independent replicates, each containing DRGs from 6–8 embryos from multiple litters, were prepared for each genotype. RNA labeling, microarray hybridization and statistical analysis were carried out by MGX-Montpellier GenomiX platform. For the preparation of the labeled Cy3- and Cy5- aRNA target, one microgram of the total RNA samples were amplified and labeled using the Amino Allyl Message Amp II aRNA Amplification Kit (Ambion; Austin, Texas, USA), according to the manufacturer's instructions. The Cy3- and Cy5-labeled aRNA samples were then added to Hybridization Buffer, hybridization component A and alignment oligo mix (Roche Nimblegen, Madison, Wisconsin), denatured at 95°C for three minutes and applied to an array on a 12×135K Nimblegen microarray slide. Each biological replicate sample (3wt+3ko for E14) was compared to the common reference twice. Hybridization was carried out at 42°C for 16 hours in the hybridization system 4 (Roche Nimblegen, Madison, Wisconsin). Hybridized slides were washed according to Nimblegen's protocols. Microarrays were immediately scanned at 1 µm resolution in both Cy3 and Cy5 channels with Innoscan900 scanner (Innopsys, Carbonne, France) using variable photo multiplier tube (PMT) settings to obtain maximal signal intensities. Nimblescan v2.5 software (Roche Nimblegen, Madison, Wisconsin) was used for feature extraction. To perform the analysis of the data, a script written in R language was used. R packages available in the BioConductor project [37] were used (R v2.12.2, bioconductor v2.7). The data were normalized using LOWESS (Locally Weighted Scatterplot Smoothing) method [38] from LIMMA package. 190 differentially expressed probes (corresponding to 140 genes) were identified by LIMMA (Linear Modeling of Microarray data) method [39], using FDR (False Discovery Rate) [21] cutoff 0.01 and fold-change of >1.5. Functional annotation to differentially expressed genes was assigned based on the Gene Ontology Project (http://www.geneontology.org/) and literature review.

### Axonal projection counts

For each marker, 8–10 sections from 4–5 different areas of thick glabrous skin (1^st^–2^nd^ hindpaw footpad) and 8–10 section of thin glabrous (skin adjacent to a footpad) were analyzed, 2 animals per genotype. At least 5 mm of skin was analyzed per each genotype.

### Cell counts and statistical analysis

For adult tissues, we adopted a strategy that has been previously validated for DRG cell counts [13]. Briefly, serial sections of thoracic or lumbar DRG were distributed on 6 slides which were subjected to different markers including the pan-neuronal marker *SCG10*. This approach allowed us to represent all counts as percentage of the total number of neurons (*SCG10^+^*). All cell counts were conducted by an individual who was blind to the genotype of the animals. 8–12 week old mice were used for analysis. The number of DRGs counted for each marker is indicated in figure legends. Statistical significance was set to p<0.05 and assessed using unpaired t-test for all statistical tests in the manuscript.

### Behavioral tests

Animals were maintained under standard housing conditions (25°C, 40% humidity, 12 h light cycles, and free access to food and water). Special effort was made to minimize the number as well as the stress and suffering of mice used in this study. All protocols are in agreement with European Union recommendations for animal experimentation. All behaviour analysis was conducted on littermate males 8–12 weeks old. Student's T-test was used for all statistical calculations. Detailed description of tests is provided in Protocol S1.

## Supporting Information

Figure S1 Generation of *TrkAC-KI* mice. (A) Diagram of the targeting strategy for generation of *TrkAC-KI* mice. Schematic structures of *TrkA (Ntrk1)* genomic locus (top), of *TrkA* locus after homologous recombination (middle) and after neo cassette excision (bottom) are shown. The extracellular part of the TrkAC chimeric protein is encoded by first 10 exons of the endogenous TrkA in order to ensure proper expression of the *TrkAC* mRNA. The transmembrane and intracellular parts of TrkAC protein are encoded by *TrkC* cDNA. An IRES allows *Cherry* expression in *TrkAC*-expressing neurons. Selection marker *neo* is flanked by *loxP* site (triangles) and is removed by crossing to a mouse expressing ubiquitous Cre recombinase. Positions of HincII restriction enzymes as well as locations of external and neo probes are shown. P1, P2, P3 are primers used in genotyping strategy of tail PCR. (B) Southern blot analysis of a positive embryonic stem cell clone used to generate *TrkAC-KI* founders. (C) Genotyping results on tail DNA from knock-in, heterozygous and wild type littermate mice using primers P1, P2 and P3 shown in (A). (D) Schematic diagram of TrkAC protein. (E) Western blot of DRG extracts from E14.5 embryos and adult mice. Both mature (140 kD) and immature (110 kD) forms of TrkA and TrkAC proteins are detected in E14.5 extracts using anti-TrkA antibody. Notice the shift in molecular weight of TrkAC, since the transmembrane and intracellular regions of TrkC are longer than that of TrkA (395 vs. 380aa). Lower molecular weight forms are also detected in homozygous and heterozygous embryos, possibly representing degraded forms of the receptor, explaining the decrease in the amount of the 140 kD form. The trace amount of this lower molecular weight form is also detected in wild type embryos. In adult wild type mice, the expression of TrkA is much lower comparing to embryonic TrkA levels. Thus, the expression of TrkAC in *TrkAC-KI* adult mice is below the detection threshold. (F) Quantification of TrkA and TrkAC expression in DRGs from E14.5 embryos. Mature form (∼140 kD) of TrkAC is present at 58.3±4.9% comparing to wild type TrkA, while the sum of all forms is equal between control and mutant mice, suggesting that expression from *Ntrk1* locus in *TrkAC-KI* mice is equivalent to that of wild type. The bar graph represents four independent experiments (mean ± s.e.m). (G) Western blot on DRG extracts from E14.5 embryos using an antibody specific to the C-terminal (intracellular) part of Trks revealed a ∼45 kD band in KI and Heterozygous samples (red arrowhead), but not in WT samples. The molecular weight of this band corresponds to the predicted size of TrkC intracellular domain suggesting an active cleavage of TrkAC receptor. (H) DRG extracts from E14.5 embryos were treated with PNGase F overnight. Western blots were performed using anti-TrkA antibodies specific to N-terminal (extracellular) and C-terminal (intracellular) parts. The lower KI-specific band detected by the N-term specific anti-TrkA antibody shifts to approximately 45 kD which corresponds to the weight of unglycosylated extracellular domain (solid blue arrowheads). This band is not detected by the C-term specific antibody. The upper KI-specific band also shifts upon PNGase F treatment, though to a lower extent (open blue arrowheads). As in (G), a ∼45 kD band, whose molecular weight is not affected by deglycosylation, is detected by the C-term specific antibody (red arrowhead).
(TIF)

Figure S2 Downstream signaling effectors are activated in dissociated DRG neurons from *TrkAC-KI* in response to NGF stimulation. (A) Western blot on DRG neuron lysates using antibodies against TrkA, pAkt, pERK(MAPK) and ERK. Before lysis, DRG neurons from E14.5 embryos were grown overnight in presence of NGF, starved for 48 h in NGF-free medium and stimulated for indicated times with 100 ng/ml NGF. (B) Quantification of TrkA amount from three independent experiments is shown. (C) Quantification of pERK activation. Despite lower levels of TrkAC protein in cultured DRG neurons from *TrkAC-KI* embryos, levels of pERK were significantly increased in TrkAC neurons after 5 and 15 min stimulation with NGF comparing to non-stimulated TrkAC neurons (p = 6×10–5 and 0.027 respectively). Moreover, ERK activation was similar between TrkAC and wild type neurons after 5 min stimulation, but lower in TrkAC comparing to wild type neurons after 15 min stimulation with NGF (p = 0.028). (D) Quantification of pAkt activation. Amount of pAkt was similar between TrkAC and wild type neurons after 5 and 15 min stimulation with NGF. However, basal level of Akt activation was higher in TrkAC neurons comparing to wild type neurons (p = 0.0002). In each independent experiment, lysates of *TrkAC-KI* and wild type cultures were analyzed in parallel. The amount of pAkt and pERK were normalized to ERK and expressed as percentage of wild type amount after 5 min stimulation. The bar graphs represent data from three independent experiments (mean ± s.e.m). **p<0.01.
(TIF)

Figure S3 Postnatal expression of nociceptive markers is normal in DRGs from TrkAC-KI mice. Expression of *TrpA1* (A,B), *TrpC3* (C,D), *TrpV1* (E,F) and *MrgprB4* (G,H) mRNAs in adult DRGs from TrkAC-KI and wild type littermates. Scale bar is 50 mm.
(TIF)

Figure S4 A number of genes normally expressed in DRGs from E14.5 and P0 *TrkAC-KI* mice. (A–F) *In situ* hybridization demonstrates that expression of *GFRα1-3* is similar between in E14.5 DRGs from *TrkAC-KI* and control embryos. (G,H) Immunostaining of Runx1 shows that this transcription factor is expressed normally in *TrkAC-KI* DRGs at E14.5. (I–N) *In situ* hybridization using probes against *GFRα1-3* shows that expression of these genes is similar between DRGs from newborn *TrkAC-KI* and control mice. (O,P) Immunostaining with anti-Runx1 antibody shows normal expression of this protein in P0 *TrkAC-KI* DRGs. Note that while expression of Runx1 is drastically downregulated and GFRα1 completely absent from NGF−/−;Bax−/− DRGs at P0 (Luo *et.al.* 2007), expression of these markers is normal in DRGs from *TrkAC-KI* mice at this stage (I,M and O,P). (Q–V) Immunostaining of DRGs from E14.5 embryos using antibodies against TrkA, TrkB, TrkC and Ret. Scale bar is 50 µm.
(TIF)

Figure S5 Peripheral innervation of hairy skin is severely decreased in *TrkAC-KI* mice. (A–D) Peptidergic (CGRP-positive) and total (PGP9.5-positive) fiber innervation is decreased in back skin from adult *TrkAC-KI* animals. Scale bar is 50 µm.
(TIF)

Figure S6 Sensory neurons from *TrkAC-KI* embryos exhibit defective NGF-dependent axonal extension *in vitro*. (A–C) DRG explants from *TrkAC-KI* and wild type embryos (E13.5) were grown in presence of 50 ng/ml NGF overnight. *TrkAC-KI* explants had significantly lower density of neurites. Data from three independent experiments, including 14 wild type and 17 *TrkAC-KI* explants, are shown (C). (D–F) There was a significantly lower number of neurite-bearing cells present in cultures from E14.5 *TrkAC-KI* embryonic DRGs. Only caspase3 negative neurons were counted in these experiments. At least 900 neurons from several embryos were counted for each genotype. (G) Interestingly, the length and morphology of those TrkAC-KI neurons that did grow neurites were similar to that of wild type (n = 50 for wild type and n = 98 for *TrkAC-KI* from at least three independent culture experiments). Sensory neurons were visualised using anti-neurofilament antibody. Scale bar is 100 µm. Data represent mean ± s.e.m. * p<0.05, ** p<0.01.
(TIF)

Figure S7 Normal locomotion and exploratory behavior in *TrkAC-KI* mice. (A) *TrkAC-KI* mice behaved similarly to wild type in Open Field (n = 10 for wild type and 8 for *TrkAC-KI.* (B) There was no difference in motor coordination between *TrkAC-KI* and wild type mice, as tested by Rotarod (n = 9 for wild type and 6 for *TrkAC-KI*). Data represent mean ± s.e.m.
(TIF)

Protocol S1 Supporting Materials and Methods.
(DOC)

Table S1 Gene expression profiling of DRGs from E14.5 WT and *TrkAC-KI* embryos.
(DOC)
