# Supplementary figures and images for: Uncoupling of Molecular Maturation from Peripheral Target Innervation in Nociceptors Expressing a Chimeric TrkA/TrkC Receptor
Source: PLoS Genet. 2014 Feb 6;10(2):e1004081. doi: 10.1371/journal.pgen.1004081 (PMC3916231; doi:10.1371/journal.pgen.1004081)

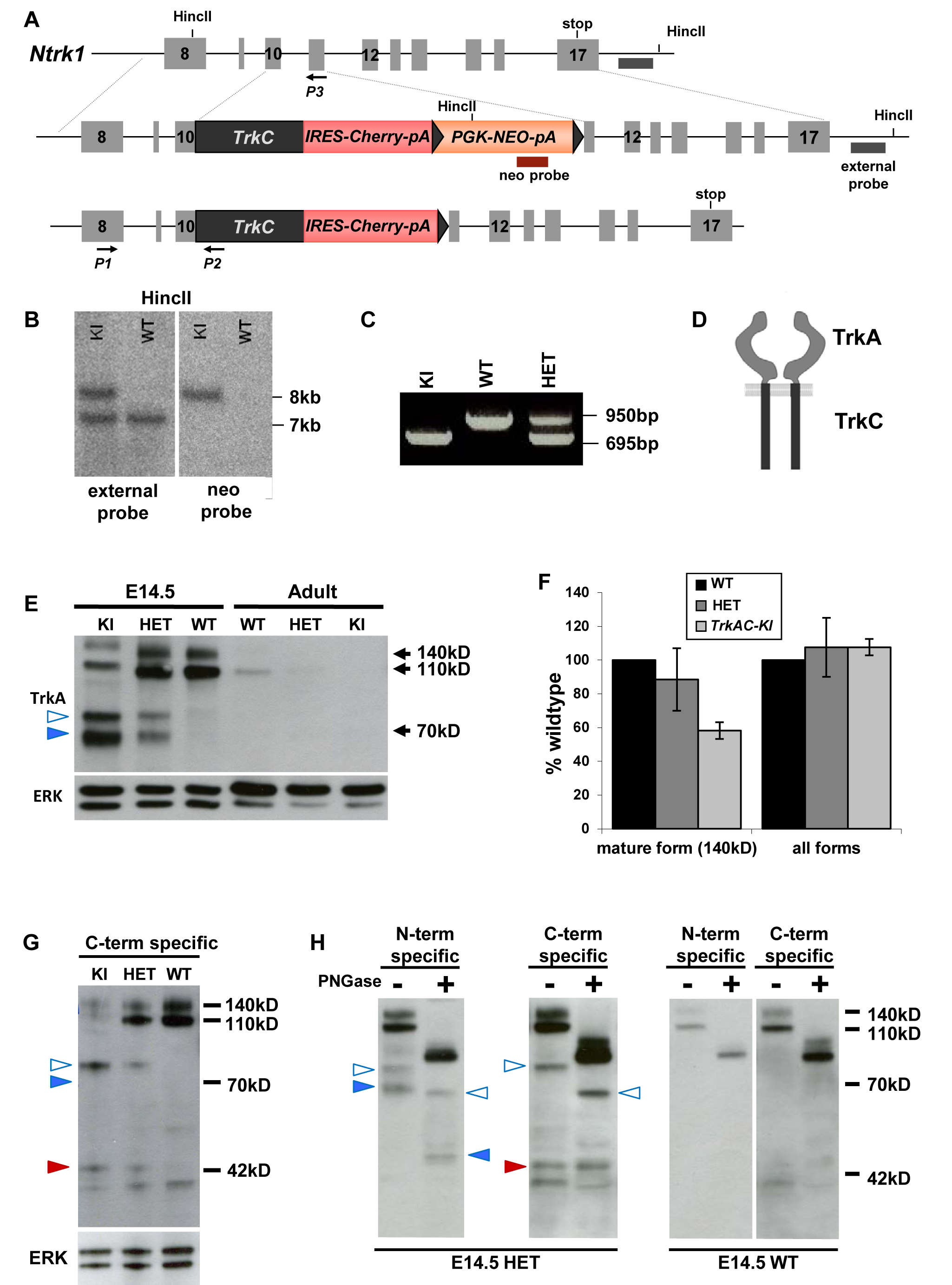

Supplement: Figure S1 — Generation of TrkAC-KI mice. (A) Diagram of the targeting strategy for generation of TrkAC-KI mice. Schematic structures of TrkA (Ntrk1) genomic locus (top), of TrkA locus after homologous recombination (middle) and after neo cassette excision (bottom) are shown. The extracellular part of the TrkAC chimeric protein is encoded by first 10 exons of the endogenous TrkA in order to ensure proper expression of the TrkAC mRNA. The transmembrane and intracellular parts of TrkAC protein are encoded by TrkC cDNA. An IRES allows Cherry expression in TrkAC-expressing neurons. Selection marker neo is flanked by loxP site (triangles) and is removed by crossing to a mouse expressing ubiquitous Cre recombinase. Positions of HincII restriction enzymes as well as locations of external and neo probes are shown. P1, P2, P3 are primers used in genotyping strategy of tail PCR. (B) Southern blot analysis of a positive embryonic stem cell clone used to generate TrkAC-KI founders. (C) Genotyping results on tail DNA from knock-in, heterozygous and wild type littermate mice using primers P1, P2 and P3 shown in (A). (D) Schematic diagram of TrkAC protein. (E) Western blot of DRG extracts from E14.5 embryos and adult mice. Both mature (140 kD) and immature (110 kD) forms of TrkA and TrkAC proteins are detected in E14.5 extracts using anti-TrkA antibody. Notice the shift in molecular weight of TrkAC, since the transmembrane and intracellular regions of TrkC are longer than that of TrkA (395 vs. 380aa). Lower molecular weight forms are also detected in homozygous and heterozygous embryos, possibly representing degraded forms of the receptor, explaining the decrease in the amount of the 140 kD form. The trace amount of this lower molecular weight form is also detected in wild type embryos. In adult wild type mice, the expression of TrkA is much lower comparing to embryonic TrkA levels. Thus, the expression of TrkAC in TrkAC-KI adult mice is below the detection threshold. (F) Quantification of Trk [file pgen.1004081.s001.tif]

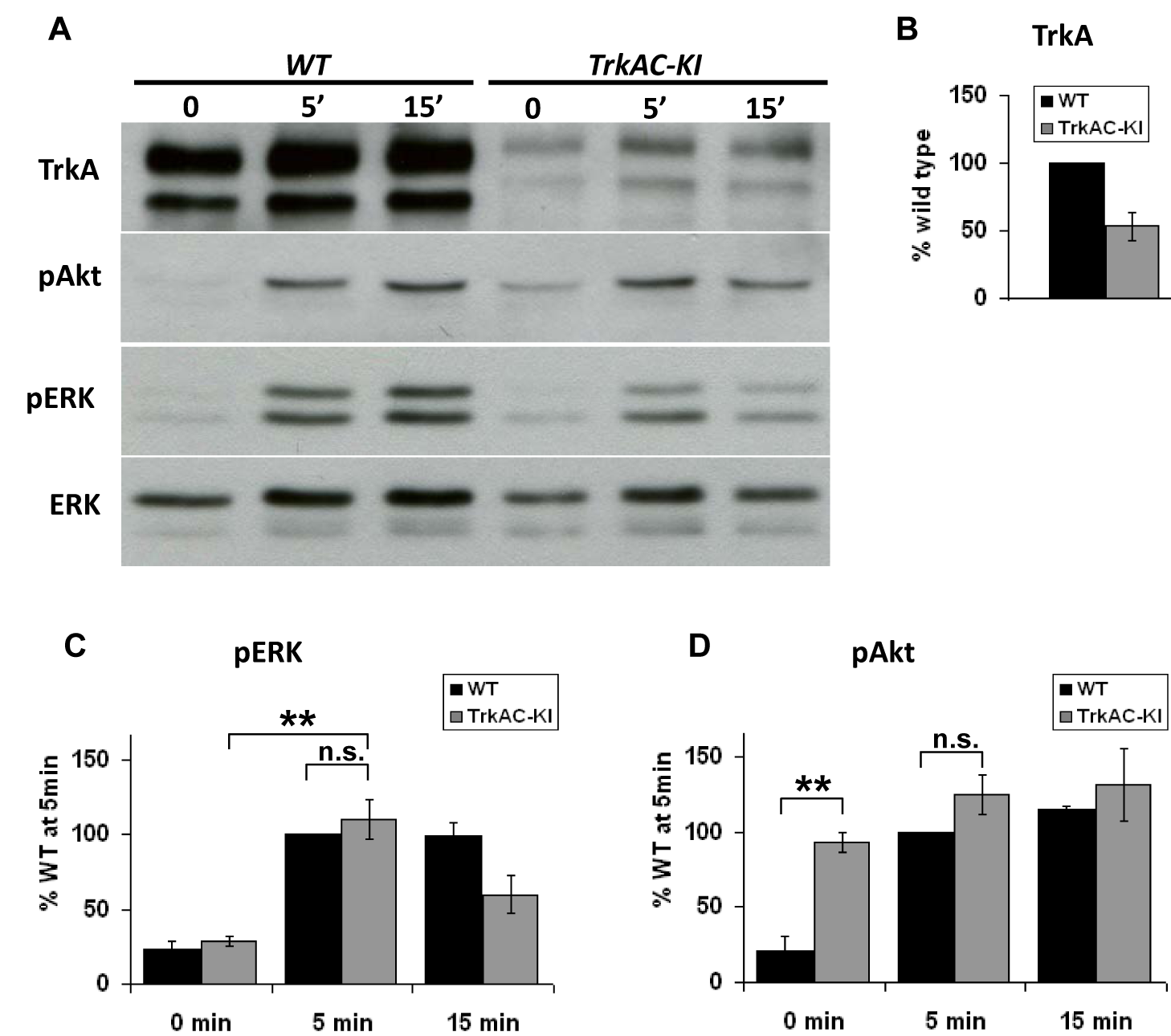

Supplement: Figure S2 — Downstream signaling effectors are activated in dissociated DRG neurons from TrkAC-KI in response to NGF stimulation. (A) Western blot on DRG neuron lysates using antibodies against TrkA, pAkt, pERK(MAPK) and ERK. Before lysis, DRG neurons from E14.5 embryos were grown overnight in presence of NGF, starved for 48 h in NGF-free medium and stimulated for indicated times with 100 ng/ml NGF. (B) Quantification of TrkA amount from three independent experiments is shown. (C) Quantification of pERK activation. Despite lower levels of TrkAC protein in cultured DRG neurons from TrkAC-KI embryos, levels of pERK were significantly increased in TrkAC neurons after 5 and 15 min stimulation with NGF comparing to non-stimulated TrkAC neurons (p = 6×10^–5^ and 0.027 respectively). Moreover, ERK activation was similar between TrkAC and wild type neurons after 5 min stimulation, but lower in TrkAC comparing to wild type neurons after 15 min stimulation with NGF (p = 0.028). (D) Quantification of pAkt activation. Amount of pAkt was similar between TrkAC and wild type neurons after 5 and 15 min stimulation with NGF. However, basal level of Akt activation was higher in TrkAC neurons comparing to wild type neurons (p = 0.0002). In each independent experiment, lysates of TrkAC-KI and wild type cultures were analyzed in parallel. The amount of pAkt and pERK were normalized to ERK and expressed as percentage of wild type amount after 5 min stimulation. The bar graphs represent data from three independent experiments (mean ± s.e.m). **p<0.01. (TIF) [file pgen.1004081.s002.tif]

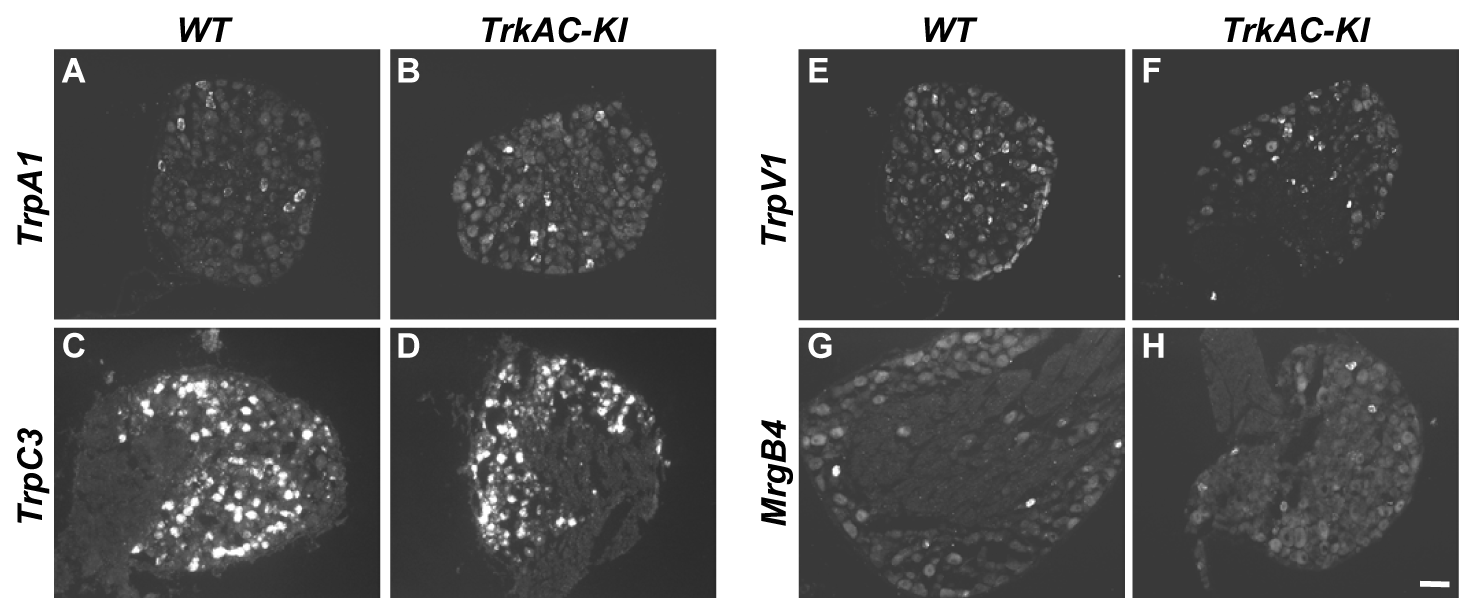

Supplement: Figure S3 — Postnatal expression of nociceptive markers is normal in DRGs from TrkAC-KI mice. Expression of TrpA1 (A,B), TrpC3 (C,D), TrpV1 (E,F) and MrgprB4 (G,H) mRNAs in adult DRGs from TrkAC-KI and wild type littermates. Scale bar is 50 mm. (TIF) [file pgen.1004081.s003.tif]

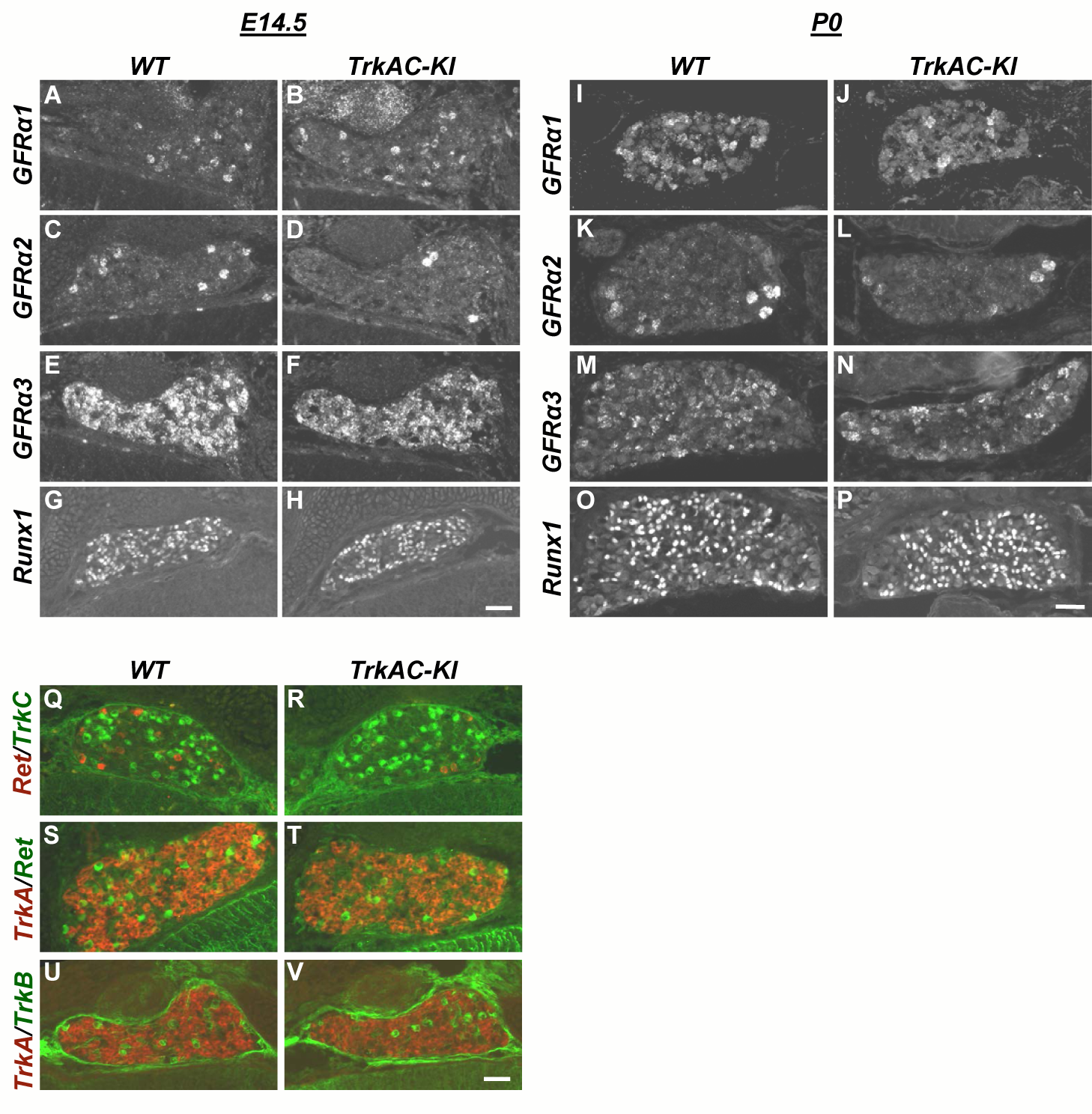

Supplement: Figure S4 — A number of genes normally expressed in DRGs from E14.5 and P0 TrkAC-KI mice. (A–F) In situ hybridization demonstrates that expression of GFRα1-3 is similar between in E14.5 DRGs from TrkAC-KI and control embryos. (G,H) Immunostaining of Runx1 shows that this transcription factor is expressed normally in TrkAC-KI DRGs at E14.5. (I–N) In situ hybridization using probes against GFRα1-3 shows that expression of these genes is similar between DRGs from newborn TrkAC-KI and control mice. (O,P) Immunostaining with anti-Runx1 antibody shows normal expression of this protein in P0 TrkAC-KI DRGs. Note that while expression of Runx1 is drastically downregulated and GFRα1 completely absent from NGF−/−;Bax−/− DRGs at P0 (Luo et.al. 2007), expression of these markers is normal in DRGs from TrkAC-KI mice at this stage (I,M and O,P). (Q–V) Immunostaining of DRGs from E14.5 embryos using antibodies against TrkA, TrkB, TrkC and Ret. Scale bar is 50 µm. (TIF) [file pgen.1004081.s004.tif]

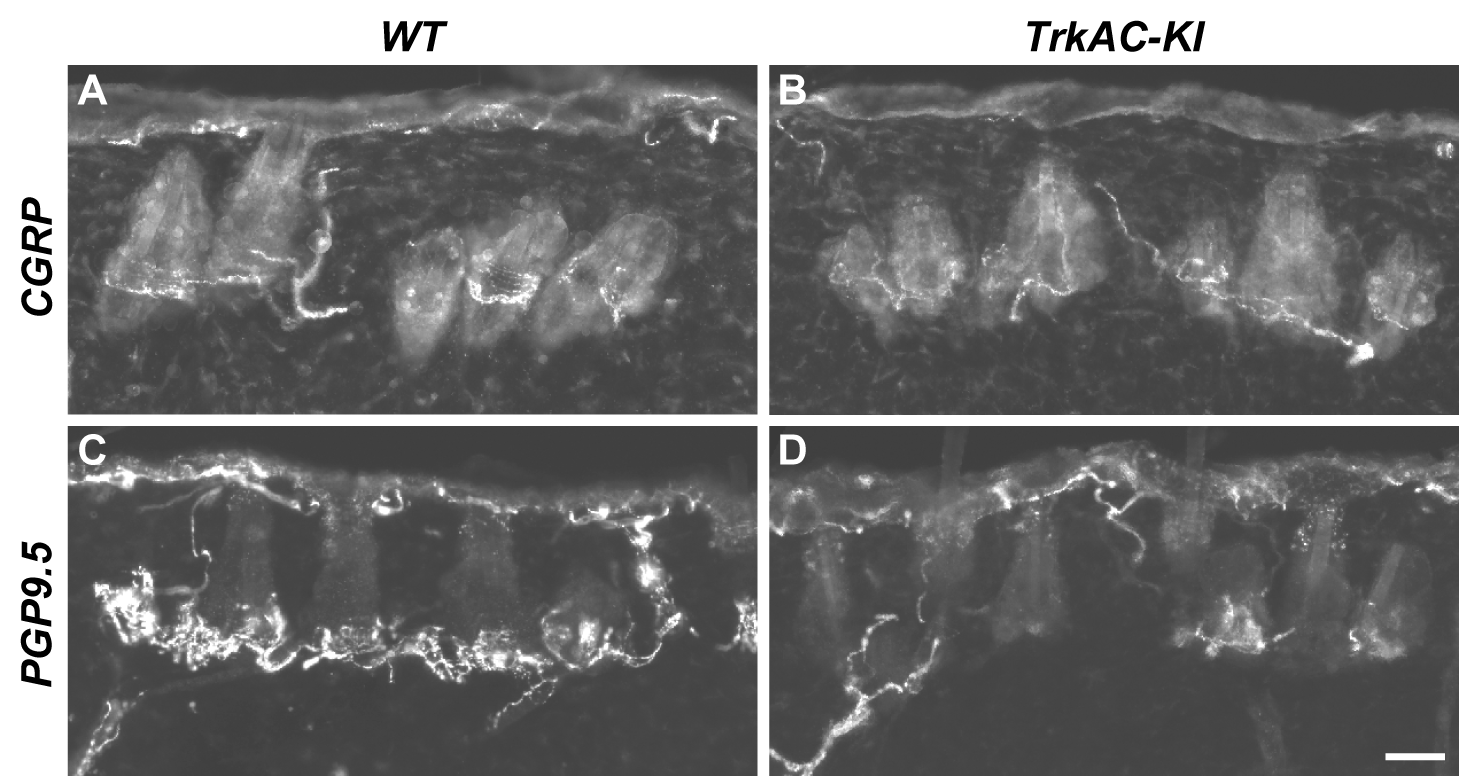

Supplement: Figure S5 — Peripheral innervation of hairy skin is severely decreased in TrkAC-KI mice. (A–D) Peptidergic (CGRP-positive) and total (PGP9.5-positive) fiber innervation is decreased in back skin from adult TrkAC-KI animals. Scale bar is 50 µm. (TIF) [file pgen.1004081.s005.tif]

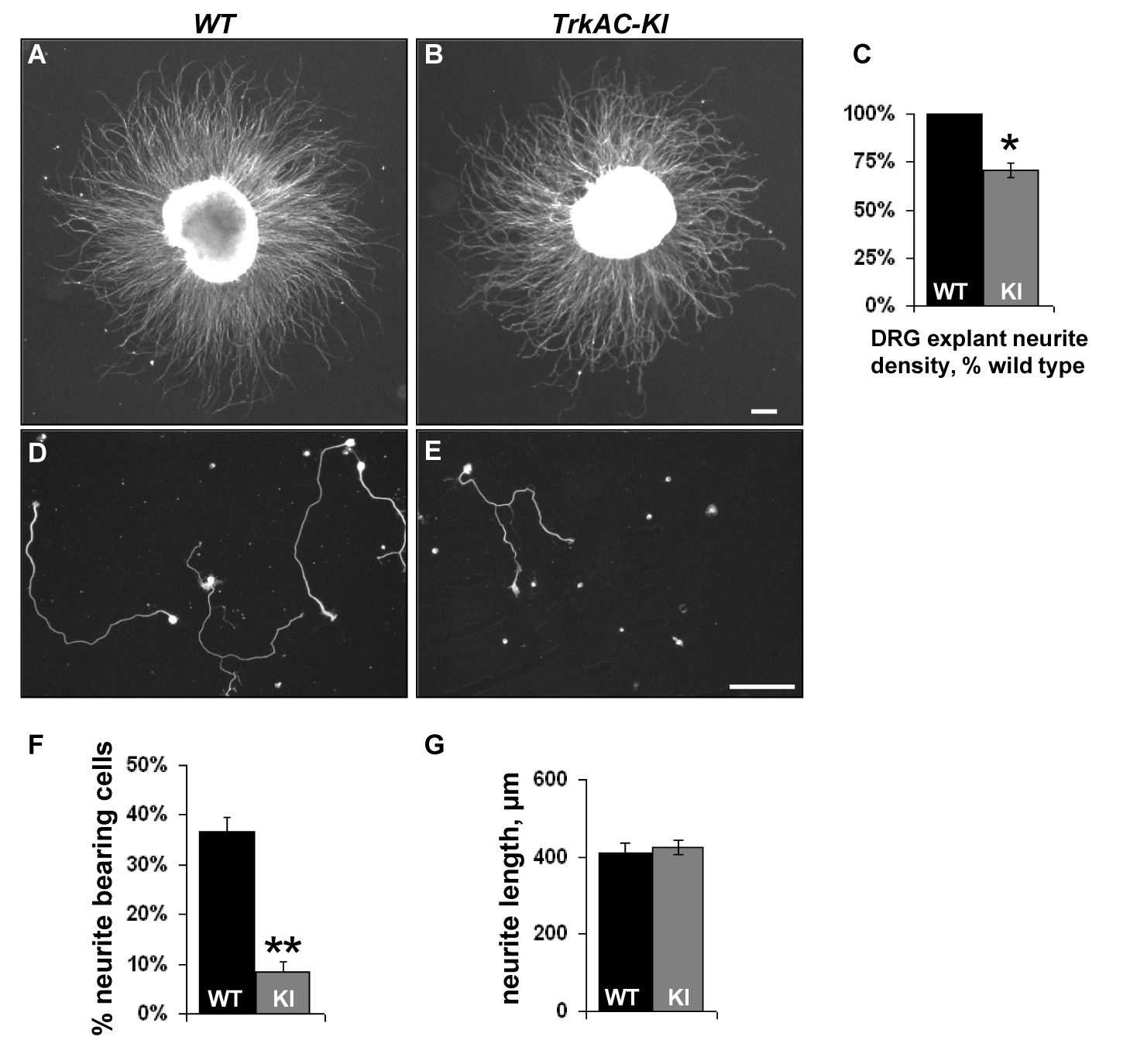

Supplement: Figure S6 — Sensory neurons from TrkAC-KI embryos exhibit defective NGF-dependent axonal extension in vitro. (A–C) DRG explants from TrkAC-KI and wild type embryos (E13.5) were grown in presence of 50 ng/ml NGF overnight. TrkAC-KI explants had significantly lower density of neurites. Data from three independent experiments, including 14 wild type and 17 TrkAC-KI explants, are shown (C). (D–F) There was a significantly lower number of neurite-bearing cells present in cultures from E14.5 TrkAC-KI embryonic DRGs. Only caspase3 negative neurons were counted in these experiments. At least 900 neurons from several embryos were counted for each genotype. (G) Interestingly, the length and morphology of those TrkAC-KI neurons that did grow neurites were similar to that of wild type (n = 50 for wild type and n = 98 for TrkAC-KI from at least three independent culture experiments). Sensory neurons were visualised using anti-neurofilament antibody. Scale bar is 100 µm. Data represent mean ± s.e.m. * p<0.05, ** p<0.01. (TIF) [file pgen.1004081.s006.tif]

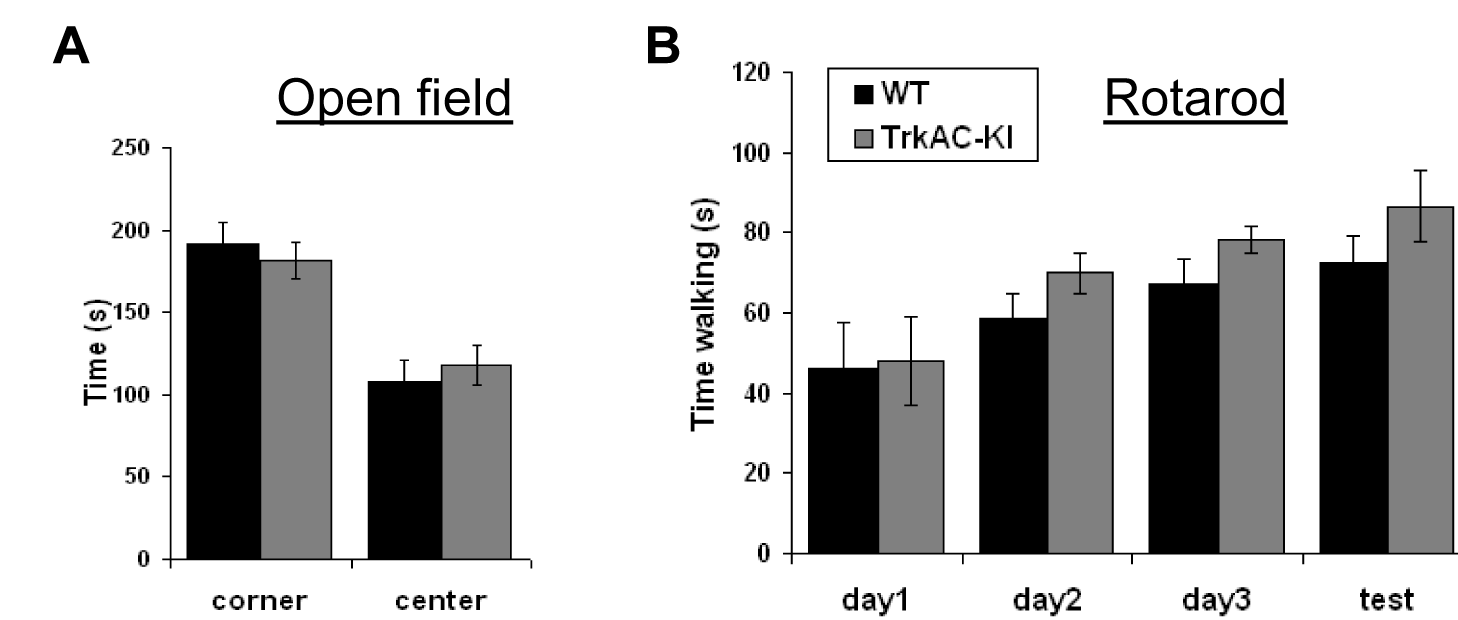

Supplement: Figure S7 — Normal locomotion and exploratory behavior in TrkAC-KI mice. (A) TrkAC-KI mice behaved similarly to wild type in Open Field (n = 10 for wild type and 8 for TrkAC-KI. (B) There was no difference in motor coordination between TrkAC-KI and wild type mice, as tested by Rotarod (n = 9 for wild type and 6 for TrkAC-KI). Data represent mean ± s.e.m. (TIF) [file pgen.1004081.s007.tif]
