# Supplementary material for: Uncoupling of Molecular Maturation from Peripheral Target Innervation in Nociceptors Expressing a Chimeric TrkA/TrkC Receptor
Source: PLoS Genet. 2014 Feb 6;10(2):e1004081. doi: 10.1371/journal.pgen.1004081 (PMC3916231; doi:10.1371/journal.pgen.1004081)
Supplement: Protocol S1 — Supporting Materials and Methods. (DOC) [file pgen.1004081.s008.doc]

**Protocol S1: Supporting materials and methods**

**Generation of TrkA^TrkAC^ (*TrkAC-KI*) knock in mice**

Targeting strategy for generating *TrkAC-KI* mice is shown in the **Supplementary Fig. 1**. The chimeric TrkAC protein encoded by the construct contains the extracellular region of TrkA (NP_001028296, amino acids 1-419) and the transmembrane and intracellular regions of TrkC (NP_032772, amino acids 431-825). The targeting construct was build in the plasmid vector pl452AM (a kind gift from A. Patapoutian), which contains *loxP-*flanked neomycin gene (*neo*) under PGK promoter. The 5’ arm of the targeting construct, consisting of 1.5kb genomic region containing exons 8-10 of *TrkA (Ntrk1)*, was amplified by PCR using primers 5’-AGTCGACATGTCTGCAGAATAATAAAACTTGTTTG-3’ 5’ ACCCCAAAAGGGGTTTCATCTTTCTTCTCC-3’ and fused to 1.2kb fragment of *TrkC (Ntrk3)* cDNA encoding transmembrane and intracellular parts of TrkC (NM_008746, nucleotides 1351-2533). The resulting DNA fragment was then fused to a cassette containing *IRES*, *Cherry* and *SV40poly(A)* sequences. The 3’ arm of the targeting construct (4.5kb) was amplified by PCR using primers 5’- AACGCGTACAGAGGAGCAAATTTGGGATCAAC-3’ and 5’-TATTATGATGGATGCTGGCCATGAAG-3’. 323bp region of *TrkA*, containing part of exon11, is deleted after homologous recombination. The targeting construct was linearized and transfected into 129/SV-derived embryonic stem cells CK35. Positive clones were identified by PCR using P4, 5’-TAGAAGACACCTTCCCTCAGTCA-3’ and P2, 5’-TAAAGAGAACCACCAGAAGGACG-3’ and confirmed by Southern blot using external and neo probes. Same strategy was used to confirm the genotype of the founders generated from a positive clone. Neo cassette was then removed by crossing the newly generated *TrkAC-KI* mice to a cre-deleter line. The absence of neo cassette was confirmed by PCR and Southern blot. A multiplex PCR genotyping strategy was designed for tail DNA using the following primers P1, 5’-GACAACCCTTTTGAGTTCAACC-3’; P2, 5’-TAAAGAGAACCACCAGAAGGACG-3’ and P3, 5’-TTGAGCACAAGAAGGAGGGCAG-3’. Animals were maintained under standard housing conditions (23°C, 40% humidity, 12h light cycles and free access to food and water). All protocols were in agreement with EU recommendations for animal experimentation.

**Behavioral tests**

All behaviour analysis was conducted on littermate males 8–12 weeks old. Animals were acclimated for at least 30 min to their testing environment prior to all experiments. Initial tests were performed in mixed 129SV/C57B6 genetic background and were then confirmed with *TrkAC-KI* mice that were backcrossed to C57B6 for 9 generations. Student t-test was used for all statistical calculations.

*Thermal Gradient test*

This test has been described previously (Moqrich et al., 2005). Briefly, mice were individually tracked for 90 min in four separate arenas of the thermal gradient apparatus (Bioseb). A controlled and stable temperature gradient of 14°C to 53.5°C was maintained using two Peltier heating/cooling devices positioned at each end of the aluminium floor. Each arena was virtually divided into 15 zones of equal size (8 cm) with a distinct and stable temperature. The tracking was performed using a video camera controlled by the software provided by the manufacturer.

*Two-temperature choice tests.*

Two mice were placed simultaneously in each plate of the two temperature choice apparatus (Bioseb). Mice were tracked for 40 min using the Bioseb software. During the first 20 min, both plates were kept at 20°C. After this period of acclimatisation, plates were rapidly adjusted to the appropriate choice temperatures. Percentage of time spent in the cooler area during the 20 min testing period was recorded for each choice test.

*Tail flick*

Mice were confined within a restraining tube and a radiant heat was applied to the tail (Bioseb). Each mouse was tested three times with a rest period of 5 min between trials. The tail withdrawal time was recorded. A cut-off of 20 sec was applied to avoid tissue damage.

*Hot plate*

Mice were placed on a plate set at 48°C. The latency to the onset of hindpaw licking or shaking was measured. Each mouse was tested two times with a resting period of 5 min between trials. After a resting period of at least 1 hr, the experiment was repeated for 50°C and 52°C. A cut-off of 60s was applied to avoid tissue damage.

*Cold plate*

Rearing time during one minute was measured on a plate cooled at 22°C, 10°C, and 4°C. Each mouse was exposed three times to each temperature with a minimum of 5 min resting period between trials and 1hr resting period between temperatures. Sensitivity to cold was also assessed by measuring the latency to the onset of hindpaw licking after mice were placed on a 4°C plate.

*CFA-induced thermal hyperalgesia (Hargreaves test) and mechanical allodynia (Dynamic Von Frey plantar test and Dynamic weight bearing test).*

10 µl of Complete Freund’s adjuvant (CFA) was injected into the plantar surface of the left hind-paw of anaesthetized mice using a Hamilton syringe. Injected paws were assessed for signs of acute inflammation, such as edema and redness, 24 hrs after injection. The responses were measured before injection (Day0), as well as one and three days after CFA injection. For Dynamic Weight Bearing, each mouse was placed into the DWB chamber (Bioseb) and video-recorded for two consecutive 5 min. Weight bearing distribution was then analysed using the Easy Scoring option of the DWB software (Bioseb). For Von Frey test, the filament was applied with an increasing strength up to 10 g during 20 s. Injected and non-injected paws were tested three times with at least 5 min pause between trials. For Hargreaves test, each mouse was placed in a clear Plexiglas compartment with the infrared source placed beneath the glass floor. Measurements were realized three times for each paw. IR source was adjusted to 20% and a cut-off of 20 s was applied to avoid tissue damage.

*Open Field*

Mice were placed individually in a square arena (40x40x35cm). Locomotor activity was recorded for 5 min using Bioseb tracking software. The time spent in the center area (20x20cm) was recorded for each mouse.

*Rotarod Test*

In order to assess motor coordination and balance, mice were placed on Rotarod apparatus (LSI Letica Scientific Instruments) at accelerating speed from 4 to 44rpm over 5min and the latency to fall off during this period was recorded. Mice were tested for 4 consecutive days. Each day, the animals were tested three times separated by at least 5 min resting period.

*Formalin Test*

10µl of 2% formalin (Fischer Scientific) was injected into the plantar surface of the right hindpaw of a restrained mouse using a Hamilton syringe. The mouse was then monitored for pain behavior (shaking, licking and biting of the injected paw) for 60 min. Time spend exhibiting these pain behaviors was recorded for the first phase (0-15 min) and the second phase (15-60 min).
