# Supplementary material for: Uncoupling of Molecular Maturation from Peripheral Target Innervation in Nociceptors Expressing a Chimeric TrkA/TrkC Receptor
Source: PLoS Genet. 2014 Feb 6;10(2):e1004081. doi: 10.1371/journal.pgen.1004081 (PMC3916231; doi:10.1371/journal.pgen.1004081)
Supplement: Table S1 — Gene expression profiling of DRGs from E14.5 WT and TrkAC-KI embryos. (DOC) [file pgen.1004081.s009.doc]

**Table S1**. Gene expression profiling of DRGs from E14.5 WT and *TrkAC-KI* embryos.

Genes with downregulated expression in E14.5 DRGs from *TrkAC-KI* embryos.

| Gene Symbol | Gene Title | Fold Change | Function | In previous screens?^19,20^ |
| --- | --- | --- | --- | --- |
| Ntrk1 | neurotrophic tyrosine kinase, receptor, type 1 | 15,79 |  |  |
| Ntrk1 | neurotrophic tyrosine kinase, receptor, type 1 | 14,41 |  |  |
| Ntrk1 | neurotrophic tyrosine kinase, receptor, type 1 | 14,21 |  |  |
| Ntrk1 | neurotrophic tyrosine kinase, receptor, type 1 | 13,72 |  |  |
| Ntrk1 | neurotrophic tyrosine kinase, receptor, type 1 | 11,34 |  |  |
| Ntrk1 | neurotrophic tyrosine kinase, receptor, type 1 | 5,67 |  |  |
| --- | --- | 3,71 |  |  |
| --- | --- | 3,57 |  |  |
| --- | --- | 3,57 |  |  |
| Rbm8a | RNA binding motif protein 8a | 3,53 | RB |  |
| --- | --- | 3,37 |  |  |
| Chodl | chondrolectin | 3,21 | CC/CA | x |
| Chodl | chondrolectin | 3,00 | CC/CA | x |
| Car3 | carbonic anhydrase 3 | 2,77 | M |  |
| Car3 | carbonic anhydrase 3 | 2,73 | M |  |
| Car3 | carbonic anhydrase 3 | 2,67 | M |  |
| Golph3l | golgi phosphoprotein 3-like | 2,59 | TR |  |
| 5730508B09Rik | RIKEN cDNA 5730508B09 gene | 2,57 | UM |  |
| --- | --- | 2,52 |  |  |
| Gm10925 | predicted gene 10925 | 2,41 | UM |  |
| Gm5469 | predicted gene 5469 | 2,29 | NC |  |
| Rbm8a | RNA binding motif protein 8a | 2,23 | RB |  |
| Tmx1 | thioredoxin-related transmembrane protein 1 | 2,21 | CRH |  |
| Lrig2 | leucine-rich repeats and immunoglobulin-like domains 2 | 2,21 | CC/CA |  |
| Arl6ip5 | ADP-ribosylation factor-like 6 interacting protein 5 | 2,14 | TR |  |
| Cartpt | CART prepropeptide | 2,13 | CC/CA |  |
| Selt | Selenoprotein T | 2,12 | CRH |  |
| Rgs4 | regulator of G-protein signaling 4 | 2,11 | IS | x |
| Rgs4 | regulator of G-protein signaling 4 | 2,06 | IS | x |
| Atp1b1 | ATPase, Na+/K+ transporting, beta 1 polypeptide | 2,05 | CC/CA |  |
| Spock3 | sparc/osteonectin, cwcv and kazal-like domains proteoglycan 3 | 2,04 | CC/CA |  |
| Cbln2 | cerebellin 2 precursor protein | 2,03 | CC/CA |  |
| --- | --- | 2,02 |  |  |
| Slc22a15 | solute carrier family 22 (organic anion/cation transporter), member 15 | 2,01 | IT |  |
| Chodl | chondrolectin | 2,01 | CC/CA | x |
| Ret | ret proto-oncogene | 2,01 | CC/CA | x |
| Alcam | activated leukocyte cell adhesion molecule | 2,00 | CC/CA |  |
| Ttc3 | tetratricopeptide repeat domain 3 | 1,99 | IS |  |
| Gspt1 | G1 to S phase transition 1 | 1,98 | TS |  |
| Ttc3 | tetratricopeptide repeat domain 3 | 1,97 | IS |  |
| Dzip3 | DAZ interacting protein 3, zinc finger | 1,97 | D |  |
| Ttc3 | tetratricopeptide repeat domain 3 | 1,96 | IS |  |
| Hbs1l | similar to Hbs1l protein; predicted gene 9923; Hbs1-like (S. cerevisiae) | 1,95 | TS |  |
| Golph3l | golgi phosphoprotein 3-like | 1,93 | TR |  |
| Ttc3 | tetratricopeptide repeat domain 3 | 1,93 | IS |  |
| Ret | ret proto-oncogene | 1,92 | CC/CA | x |
| Tspan13 | tetraspanin 13 | 1,90 | UM |  |
| Polr3gl | polymerase (RNA) III (DNA directed) polypeptide G like | 1,88 | T/CB |  |
| Ttc3 | tetratricopeptide repeat domain 3 | 1,86 | IS |  |
| B3galnt1 | UDP-GalNAc:betaGlcNAc beta 1,3-galactosaminyltransferase, polypeptide 1 | 1,85 | PTM |  |
| Tmem49 | transmembrane protein 49 VMP1 promotes vacuole formation | 1,85 | UM |  |
| Tmem59 | transmembrane protein 59 glycosylation/maturation of APP, BACE | 1,84 | PTM |  |
| Emb | embigin | 1,84 | CC/CA |  |
| Ttc3 | tetratricopeptide repeat domain 3 | 1,83 | IS |  |
| Atrnl1 | attractin like 1 | 1,83 | CC/CA |  |
| Evi5 | ecotropic viral integration site 5 | 1,82 | C |  |
| Atp1b1 | ATPase, Na+/K+ transporting, beta 1 polypeptide | 1,82 | CC/CA |  |
| Ttc3 | tetratricopeptide repeat domain 3 | 1,80 | IS |  |
| Rtn4 | reticulon 4 | 1,80 | TR |  |
| Tmed9 | transmembrane emp24 protein transport domain containing 9 | 1,80 | TR |  |
| Dld | dihydrolipoamide dehydrogenase | 1,80 | CRH |  |
| Fam171b | family with sequence similarity 171, member B | 1,79 | UM |  |
| Mapk8 | mitogen-activated protein kinase 8 | 1,79 | IS | x |
| Gm9797 | predicted pseudogene 9797 | 1,79 | NC |  |
| Selt | Selenoprotein T | 1,78 | CRH |  |
| Phtf1 | putative homeodomain transcription factor 1 | 1,78 | T/CB |  |
| Gm7308 | predicted pseudogene 7308 | 1,78 | NC |  |
| Nap1l1 | nucleosome assembly protein 1-like 1 | 1,78 | T/CB |  |
| Gm3380 | predicted gene 3380 | 1,77 | UM |  |
| Atp2c1 | ATPase, Ca++-sequestering | 1,77 | IT |  |
| Pvrl3 | poliovirus receptor-related 3 | 1,77 | CC/CA |  |
| Tspan6 | tetraspanin 6 | 1,77 | UM |  |
| Cd200 | CD200 antigen | 1,77 | CC/CA |  |
| Dera | 2-deoxyribose-5-phosphate aldolase homolog (C. elegans) | 1,76 | M |  |
| Rbm9 | RNA binding motif protein 9 | 1,76 | RB |  |
| Clcn4-2 | chloride channel 4-2 | 1,76 | IT |  |
| Atp1b1 | ATPase, Na+/K+ transporting, beta 1 polypeptide | 1,75 | CC/CA |  |
| Pcolce2 | procollagen C-endopeptidase enhancer 2 | 1,75 | CC/CA |  |
| Pcdha1 | Protocadherin 1 | 1,75 | CC/CA |  |
| Serpina3n | serine (or cysteine) peptidase inhibitor, clade A, member 3N | 1,75 | CC/CA |  |
| Actr2 | ARP2 actin-related protein 2 homolog (yeast) | 1,74 | C |  |
| Laptm4b | lysosomal-associated protein transmembrane 4B | 1,74 | D |  |
| Fstl5 | follistatin-like 5 | 1,73 | CC/CA | x |
| Elavl2 | ELAV (embryonic lethal, abnormal vision, Drosophila)-like 2 (Hu antigen B) | 1,73 | RB | x |
| Ggh | gamma-glutamyl hydrolase | 1,72 | M |  |
| Gpm6b | glycoprotein m6b | 1,71 | CC/CA |  |
| Tmeff2 | transmembrane protein with EGF-like and two follistatin-like domains 2 | 1,71 | CC/CA |  |
| Sar1b | SAR1 gene homolog B (S. cerevisiae) | 1,70 | TR |  |
| Ptn | pleiotrophin | 1,70 | CC/CA |  |
| Cd44 | CD44 antigen | 1,70 | CC/CA |  |
| Clcn4-2 | chloride channel 4-2 | 1,70 | IT |  |
| Rbms3 | RNA binding motif, single stranded interacting protein | 1,70 | RB |  |
| Gm7271 | predicted gene 7271 | 1,69 | NC |  |
| Sc4mol | sterol-C4-methyl oxidase-like | 1,69 | M | x |
| Lpgat1 | lysophosphatidylglycerol acyltransferase 1 | 1,69 | M |  |
| Ormdl1 | ORM1-like 1 (S. cerevisiae) | 1,69 | PTM |  |
| Emb | embigin | 1,69 | CC/CA |  |
| Bend6 | BEN domain containing 6 | 1,69 | T/CB |  |
| Rtn1 | reticulon 1 | 1,68 | TR |  |
| AF067063 | cDNA sequence AF067063 | 1,68 | U |  |
| Cnih | cornichon homolog (Drosophila) | 1,67 | TR |  |
| Fstl5 | follistatin-like 5 | 1,67 | CC/CA | x |
| Cntn1 | contactin 1 | 1,66 | CC/CA | x |
| Ttc3 | tetratricopeptide repeat domain 3 | 1,65 | IS |  |
| Tmem50a | transmembrane protein 50A | 1,65 | UM |  |
| Gpr37 | G protein-coupled receptor 37 | 1,65 | CC/CA |  |
| Tusc3 | tumor suppressor candidate 3 | 1,65 | PTM |  |
| Canx | calnexin | 1,65 | TR |  |
| --- | --- | 1,64 |  |  |
| Gm7271 | predicted gene 7271 | 1,64 | NC |  |
| Opcml | opioid binding protein/cell adhesion molecule-like | 1,64 | CC/CA |  |
| --- | --- | 1,64 |  |  |
| Art3 | ADP-ribosyltransferase 3 | 1,63 | PTM |  |
| Gm15365 | predicted gene 15365 | 1,63 | NC |  |
| Gria4 | glutamate receptor, ionotropic, AMPA4 (alpha 4) | 1,63 | CC/CA |  |
| Ntrk2 | neurotrophic tyrosine kinase, receptor, type 2 | 1,62 | CC/CA | x |
| Elovl6 | ELOVL family member 6, elongation of long chain fatty acids (yeast) | 1,62 | M |  |
| Tmeff1 | transmembrane protein with EGF-like and two follistatin-like domains 1 | 1,62 | CC/CA |  |
| Elavl2 | ELAV (embryonic lethal, abnormal vision, Drosophila)-like 2 (Hu antigen B) | 1,62 | RB | x |
| Zdhhc2 | zinc finger, DHHC domain containing 2 | 1,62 | PTM |  |
| Laptm4b | lysosomal-associated protein transmembrane 4B | 1,61 | D |  |
| Atp6v1a | ATPase, H+ transporting, lysosomal V1 subunit A | 1,61 | D |  |
| Fbxo9 | f-box protein 9 | 1,61 | D |  |
| Nap1l1 | nucleosome assembly protein 1-like 1 | 1,61 | T/CB |  |
| 0610007C21Rik | RIKEN cDNA 0610007C21 gene | 1,61 | CC/CA |  |
| Ntrk2 | neurotrophic tyrosine kinase, receptor, type 2 | 1,61 | CC/CA | x |
| Fam18b | family with sequence similarity 18, member B | 1,60 | UM |  |
| Arl6ip1 | ADP-ribosylation factor-like 6 interacting protein 1 | 1,60 | TR |  |
| Tsta3 | tissue specific transplantation antigen P35B | 1,60 | PTM |  |
| Asph | aspartate-beta-hydroxylase | 1,60 | PTM |  |
| 0610007C21Rik | RIKEN cDNA 0610007C21 gene | 1,60 | CC/CA |  |
| Gde1 | glycerophosphodiester phosphodiesterase 1 | 1,60 | M |  |
| Tmco1 | transmembrane and coiled-coil domains 1 | 1,60 | UM |  |
| Anp32e | acidic (leucine-rich) nuclear phosphoprotein 32 family, member E | 1,59 | IS |  |
| Kirrel | kin of IRRE like (Drosophila) | 1,59 | CC/CA |  |
| Syt4 | synaptotagmin IV | 1,59 | TR | x |
| Actr6 | ARP6 actin-related protein 6 homolog (yeast) | 1,58 | C |  |
| Evi5 | ecotropic viral integration site 5 | 1,58 | C |  |
| Cntn1 | contactin 1 | 1,58 | CC/CA | x |
| Kcnab1 | potassium voltage-gated channel, shaker-related subfamily, beta member 1 | 1,58 | IT |  |
| Spock3 | sparc/osteonectin, cwcv and kazal-like domains proteoglycan 3 | 1,58 | CC/CA |  |
| Tmem29 | transmembrane protein 29 | 1,58 | UM |  |
| 0610007C21Rik | RIKEN cDNA 0610007C21 gene | 1,58 | CC/CA |  |
| Ttc3 | tetratricopeptide repeat domain 3 | 1,57 | IS |  |
| Nrsn1 | neurensin 1 | 1,57 | TR |  |
| Reep3 | receptor accessory protein 3 | 1,57 | TR |  |
| Spcs3 | signal peptidase complex subunit 3 homolog | 1,57 | PTM |  |
| Itm2b | integral membrane protein 2B | 1,57 | CC/CA |  |
| Gpm6a | glycoprotein m6a | 1,57 | CC/CA |  |
| Tmem208 | transmembrane protein 208 | 1,56 | UM |  |
| Ppt1 | palmitoyl-protein thioesterase 1 | 1,56 | D |  |
| Cbln2 | cerebellin 2 precursor protein | 1,56 | CC/CA |  |
| Pcdha4 | Protocadherin 4 | 1,55 | CC/CA |  |
| Tspan18 | tetraspanin 18 | 1,54 | UM |  |
| Chp | calcium-binding protein p22 SLC9A1BP | 1,53 | TR |  |
| Tmem42 | transmembrane protein 42 | 1,53 | UM |  |
| Rtn1 | reticulon 1 | 1,53 | TR |  |
| Luzp2 | leucine zipper protein 2 | 1,53 | T/CB |  |
| Kras | v-Ki-ras2 Kirsten rat sarcoma viral oncogene homolog | 1,52 | IS |  |
| Kif5c | kinesin family member 5C | 1,52 | TR | x |
| Cd47 | CD47 antigen (Rh-related antigen, integrin-associated signal transducer) | 1,52 | CC/CA |  |
| Gm11578 | lix1 pseudogene/homolog chr1 | 1,52 | T/CB |  |
| Ttc3 | tetratricopeptide repeat domain 3 | 1,52 | IS |  |
| Cntn4 | contactin 4 | 1,52 | CC/CA | x |
| Htr7 | 5-hydroxytryptamine (serotonin) receptor 7 | 1,51 | CC/CA |  |
| Trappc4 | trafficking protein particle complex 4 | 1,51 | TR | x |
| Tgoln1 | trans-golgi network protein 2; trans-golgi network protein | 1,51 | TR |  |
| Jkamp | JNK1/MAPK8-associated membrane protein | 1,50 | IS |  |
| Arl6ip5 | ADP-ribosylation factor-like 6 interacting protein 5 | 1,50 | TR |  |

Genes with upregulated expression in E14.5 DRGs from *TrkAC-KI* embryos.

| Gene Symbol | Gene Title | Fold Change | Function | In previous screens?^19,20^ |
| --- | --- | --- | --- | --- |
| Gm15432 | predicted gene 15432 | 9,99 | UM |  |
| Gm2036 | predicted gene 2036 | 5,11 | UM |  |
| Gm2036 | predicted gene 2036 | 2,80 | UM |  |
| Arhgef11 | Rho guanine nucleotide exchange factor (GEF) 11 | 2,45 | C |  |
| Tifa | TRAF-interacting protein with forkhead-associated domain | 1,99 | D |  |
| Tbrg3 | transforming growth factor beta regulated gene 3 | 1,97 | NC |  |
| B3galnt1 | UDP-GalNAc:betaGlcNAc beta 1,3-galactosaminyltransferase, polypeptide 1 | 1,96 | PTM |  |
| A930005H10Rik | RIKEN cDNA A930005H10 gene | 1,94 | U |  |
| Pisd-ps3 | phosphatidylserine decarboxylase, pseudogene 3 | 1,92 | NC |  |
| 4933409K07Rik | RIKEN cDNA 4933409K07 gene | 1,86 | U |  |
| --- | --- | 1,81 |  |  |
| --- | --- | 1,80 |  |  |
| Rbm8a | RNA binding motif protein 8a; RIKEN cDNA B020018G12 gene | 1,80 | RB |  |
| Slc22a15 | solute carrier family 22 (organic anion/cation transporter), member 15 | 1,77 | IT |  |
| Ank3 | ankyrin 3, epithelial | 1,72 | C | x |
| Pisd-ps3 | phosphatidylserine decarboxylase, pseudogene 3 | 1,63 | NC |  |
| Suv420h2 | suppressor of variegation 4-20 homolog 2 (Drosophila) | 1,60 | T/CB |  |
| Epn2 | epsin 2 | 1,60 | TR |  |
| Add2 | adducin 2 (beta) | 1,54 | C |  |
| Atf7ip | activating transcription factor 7 interacting protein | 1,52 | T/CB |  |
| Hp1bp3 | heterochromatin protein 1, binding protein 3 | 1,50 | T/CB |  |
|  |  |  |  |  |

CC/CA cell-cell signaling/cell adhesion

TR trafficking

T/CB transcription/chromatin binding

PTM post-translational modification

M metabolism

C cytoskeleton

D degradation

IS intracellular signaling

CRH cell redox homeostasis

IT ion transport

RB RNA binding

TS translation

U unknown

UM unknown membrane

NC non-coding
